# Genetic contribution to lipid levels in early life based on 158 loci validated in adults: the FAMILY study

**DOI:** 10.1038/s41598-017-00102-1

**Published:** 2017-03-06

**Authors:** Shanice Christie, Sébastien Robiou-du-Pont, Sonia S. Anand, Katherine M. Morrison, Sarah D. McDonald, Guillaume Paré, Stephanie A. Atkinson, Koon K. Teo, David Meyre

**Affiliations:** 1 Department of Clinical Epidemiology and Biostatistics, McMaster University, Hamilton, Ontario Canada; 2 Department of Medicine, McMaster University, Hamilton, Ontario Canada; 3 Department of Pediatrics, Hamilton Health Sciences and McMaster University, Hamilton, Ontario Canada; 4 Department of Obstetrics and Gynecology, McMaster University, Hamilton, Ontario Canada; 5 Department of Pathology and Molecular Medicine, McMaster University, Hamilton, Ontario Canada

## Abstract

The contribution of polymorphisms associated with adult lipids in early life is unknown. We studied 158 adult lipid polymorphisms in 1440 participants (544 children, 544 mothers and 324 fathers) of the Family Atherosclerosis Monitoring In early life (FAMILY) birth cohort. Total cholesterol (TC), high-density lipoprotein cholesterol (HDL-C), low-density lipoprotein cholesterol (LDL-C) and triglycerides (TG) measurements were collected at birth, 3 and 5 years of age. Polymorphisms were genotyped using the Illumina Cardio-Metabochip array. Genotype scores (GS) were calculated for TC, HDL-C, LDL-C and TG. Linear and mixed-effects regressions adjusted for sex, age and population stratification were performed. The GS was associated with LDL-C level at 3 and 5 years (β = 0.017 ± 0.003, *P* = 2.9 × 10^−8^; β = 0.020 ± 0.003, *P* = 5.7 × 10^−9^) and from birth to 5 years (β = 0.013 ± 0.003, *P* = 2.6 × 10^−7^). The GS was associated with TC level at 3 and 5 years (β = 0.009 ± 0.002, *P* = 9.1 × 10^−7^; β = 0.009 ± 0.002, *P* = 7.7 × 10^−6^). *CETP* rs3764261 was associated with the HDL-C level from birth to 5 years (β = 0.064 ± 0.014, *P* =  7.4 × 10^−6^). *AMPD3* rs2923084 was associated with the HDL-C level at 5 years (β = 0.096 ± 0.024, *P* = 9.7 × 10^−5^). Known loci associated with blood lipids in adults are associated with TC, LDL-C and HDL-C, but not TG in early life. Genetically predisposed children may benefit from early lipid lowering preventative strategies.

## Introduction

Cardiovascular disease (CVD) accounts for nearly 50% of non-communicable diseases globally with 17.3 million deaths per year, a number that is expected to grow to 23.6 million by 2030^1, 2^. Abnormal lipids is one of the nine modifiable risk factors for CVD in addition to smoking, hypertension, diabetes, abdominal obesity, psychosocial factors, poor diet, alcohol consumption and lack of physical activity^3, 4^. Collectively, these risk factors account for 90% of the population attributable risk for CVD^3, 4^. On a genetic level, heritability estimates for serum lipid levels have been found to range from 28% to 78%^5, 6^. Mutations in several genes have been involved in Mendelian lipid disorders^7^. Genome-wide association studies (GWAS) have identified about 185 genetic regions affecting blood lipid levels: low density lipoprotein cholesterol (LDL-C), high-density lipoprotein cholesterol (HDL-C), total cholesterol (TC), and triglycerides (TG) in adult populations of European ancestry^8^. Recently, Mendelian randomization studies have shown lipids such as TG and LDL-C are causal determinants of CVD^9–11^. Understanding the etiology of hyperlipidemia is therefore important to modulate lipid levels and to achieve more effective CVD prevention programs during the life course.

GWAS for lipid levels have not been performed in pediatric populations to date and only one post-GWAS longitudinal study has investigated the association of lipid loci in children of Northern European ancestry^12^. Tikkanen and colleagues found GWAS lipid loci previously identified in adults were also associated with lipid levels in children and adolescents and explained more than twice the lipid variation in children compared with adults^12^. This lack of literature on lipid levels of children prompted us to investigate the fetal contributions of 158 GWAS associated-SNPs for lipids among 544 children and 896 parents (544 mothers and 324 fathers) from the Family Atherosclerosis Monitoring In earLY life (FAMILY) study.

## Results

The phenotypic characteristics of the study population are summarized in Table 1. Linear regressions and a linear mixed-effect model were used to assess the effects of the child’s SNPs (Supplementary Tables 3–6) on four lipids levels. It should be noted that no statistically significant associations were found between SNPs/GS and TG levels after the Bonferroni correction. All the statistically significant and nominal associations of child’s SNPs/GS with TC, LDL-C, HDL-C, and TG are available in Tables 2 and 3.

**Table 1:** Phenotypic information of subjects.

sex (%male)		Children (N = 544)	
		49.63%	
trait	year	mean	s.d
age	0	0.007	0.012
	3	3.088	0.166
	5	5.121	0.195
BMI	0	13.961	1.362
	3	16.212	1.241
	5	15.908	1.618
TC	0	1.635	0.414
	3	4.049	0.703
	5	4.098	0.702
LDL-C	0	0.677	0.262
	3	2.420	0.636
	5	2.346	0.662
HDL-C	0	0.795	0.277
	3	1.324	0.291
	5	1.437	0.321
TG	0	0.370	0.210
	3	0.670	0.265
	5	0.703	0.312

BMI: Body mass index. S.D: standard deviation.

**Table 2:** Results for genotype score and lipids levels.

Time of Measurement			Birth (N = 456)			3Y (N = 421)			5Y (N = 361)			Mixed Model Age Adjusted		
Effect	Trait	SNP	Beta	SE	*P*	Beta	SE	*P*	Beta	SE	*P*	Beta	SE	*P*
Child	LDL	GS	0.007	0.004	6.77 × 10^−2^	**0.017**	**0.003**	**2.94** × **10** ^−**8**^	**0.020**	**0.003**	**5.72** × **10** ^−**9**^	**0.013**	**0.003**	**2.59** × **10** ^−**7**^
Child	TC	GS	−0.0004	0.002	8.44 × 10^−1^	**0.009**	**0.002**	**9.12** × **10** ^−**7**^	**0.009**	**0.002**	**7.71** × **10** ^−**6**^	0.005	0.002	3.13 × 10^−3^
Child	HDL	GS	0,006	0,003	2,15 × 10^−2^	0,003	0,002	1,59 × 10^−1^	0,004	0,002	1,26 × 10^−1^	0,005	0,002	3,27 × 10^−3^
Child	TG	GS	0,0001	0,007	9,86 × 10^−1^	0,010	0,005	4,37 × 10^−2^	0,014	0,006	1,06 × 10^−2^	0,008	0,004	3,08 × 10^−2^

Beta and SE at birth, 3 year and 5 year are natural logarithm of the lipid level trait in mg/dl. The unit of beta and SE after linear mixed model is natural logarithm of the lipid level trait in mg/dl per year.

**Table 3:** Summary results for statistically nominal and significant associations between SNPs and lipids.

Gene	SNP	Birth (N = 456)			3y (N = 421)			5y (N = 361)			Mixed Model Age Adjusted		
		Beta	SE	p-value	Beta	SE	p-value	Beta	SE	p-value	Beta	SE	p-value
**LDL**													
*ANGPTL3*	rs2131925	0.020	0.025	4.30 × 10^−1^	0.043	0.021	**4.31** × **10** ^−**2**^	0.041	0.023	8.36 × 10^−2^	0.029	0.018	1.01 × 10^−1^
*LDLRAP1*	rs12027135	−0.017	0.022	4.45 × 10^−1^	0.011	0.019	5.45 × 10^−1^	0.046	0.021	**2.74** × **10** ^−**2**^	0.009	0.016	5.70 × 10^−1^
*ABCG5*	rs6756629	−0.017	0.049	7.34 × 10^−1^	0.102	0.041	**1.32** × **10** ^−**2**^	0.113	0.044	**1.01** × **10** ^−**2**^	0.073	0.034	2.94 × 10^−2^
*CMTM6*	rs7640978	0.030	0.043	4.89 × 10^−1^	0.022	0.037	5.61 × 10^−1^	0.083	0.040	**3.98** × **10** ^−**2**^	0.050	0.030	9.72 × 10^−2^
*DOK7*	rs6831256	0.037	0.024	1.15 × 10^−1^	0.061	0.019	**1.62** × **10** ^−**3**^	0.035	0.021	1.04 × 10^−1^	0.030	0.016	6.96 × 10^−2^
*HMGCR*	rs12916	0.022	0.024	3.59 × 10^−1^	−0.045	0.019	**1.80** × **10** ^−**2**^	0.018	0.021	3.92 × 10^−1^	0.009	0.016	5.90 × 10^−1^
*TIMD4*	rs6882076	0.002	0.024	9.26 × 10^−1^	0.049	0.020	**1.22** × **10** ^−**2**^	0.057	0.021	8.29 × 10^−3^	0.028	0.016	8.66 × 10^−2^
*FRK*	rs3798236	−0.040	0.025	1.14 × 10^−1^	−0.065	0.020	**1.30** × **10** ^−**3**^	0.013	0.022	5.52 × 10^−1^	−0.013	0.018	4.60 × 10^−1^
*MYLIP/GMPR*	rs2327951	0.000	0.027	9.93 × 10^−1^	0.061	0.022	**6.47** × **10** ^−**3**^	0.051	0.025	3.79 × 10^−2^	0.026	0.019	1.66 × 10^−1^
*ABO*	rs635634	−0.018	0.030	5.39 × 10^−1^	0.053	0.025	**3.52** × **10** ^−**2**^	0.062	0.026	1.97 × 10^−2^	0.017	0.020	4.16 × 10^−1^
*BUD13*	rs12292921	−0.048	0.051	3.44 × 10^−1^	−0.095	0.043	**2.72** × **10** ^−**2**^	−0.077	0.048	1.09 × 10^−1^	−0.078	0.035	**2.75** × **10** ^−**2**^
*HNF1A*	rs1169288	0.011	0.026	6.74 × 10^−1^	−0.041	0.020	**3.88** × **10** ^−**2**^	−0.008	0.021	7.12 × 10^−1^	0.005	0.018	7.79 × 10^−1^
*NYNRIN*	rs8017377	−0.029	0.023	2.12 × 10^−1^	0.048	0.019	**1.22** × **10** ^−**2**^	0.042	0.020	3.89 × 10^−2^	0.006	0.016	7.08 × 10^−1^
*DLG4*	rs314253	0.003	0.026	8.91 × 10^−1^	0.054	0.021	**1.05** × **10** ^−**2**^	0.032	0.023	1.73 × 10^−1^	0.030	0.018	9.21 × 10^−2^
*C17orf157*	rs12452315	0.055	0.023	1.85 × 10^−2^	0.010	0.020	6.14 × 10^−1^	0.027	0.022	2.18 × 10^−1^	0.034	0.016	**3.59** × **10** ^−**2**^
*CILP2*	rs10401969	0.030	0.048	5.26 × 10^−1^	0.088	0.038	**2.11** × **10** ^−**2**^	0.045	0.045	3.18 × 10^−1^	0.049	0.033	1.36 × 10^−1^
*LDLR*	rs6511720	0.052	0.035	1.42 × 10^−1^	0.044	0.029	1.31 × 10^−1^	0.063	0.032	5.50 × 10^−2^	0.057	0.024	**1.80** × **10** ^−**2**^
*TOMM40/APOE/APOC1*	rs157580	0.076	0.024	**1.54** × **10** ^−**3**^	0.031	0.020	1.31 × 10^−1^	0.008	0.022	7.10 × 10^−1^	0.039	0.017	**1.86** × **10** ^−**2**^
*MAFB*	rs2902940	0.023	0.025	3.76 × 10^−1^	0.066	0.020	**7.63** × **10** ^−**4**^	0.025	0.021	2.45 × 10^−1^	0.021	0.017	2.33 × 10^−1^
*SPTLC3*	rs364585	−0.001	0.025	9.73 × 10^−1^	0.045	0.021	**2.87** × **10** ^−**2**^	0.024	0.023	2.87 × 10^−1^	0.023	0.017	1.73 × 10^−1^
*GS*	GS	0.007	0.004	7.09 × 10^−2^	0.019	0.003	**2.40** × **10** ^−**9**^	0.020	0.003	2.56 × 10^−9^	0.013	0.003	**2.82** × **10** ^−**7**^
**TOTAL CHOLESTEROL**													
*EVI5*	rs6603981	−0.043	0.019	**2.74** × **10** ^−**2**^	0.008	0.015	6.02 × 10^−1^	0.013	0.016	4.06 × 10^−1^	−0.021	0.013	1.18 × 10^−1^
*PCSK9*	rs2479409	0.023	0.016	1.56 × 10^−1^	0.027	0.013	**3.55** × **10** ^−**2**^	0.005	0.013	6.78 × 10^−1^	0.013	0.011	2.19 × 10^−1^
*ABCG5*	rs6756629	0.010	0.032	7.41 × 10^−1^	0.059	0.026	**2.57** × **10** ^−**2**^	0.059	0.026	**2.32** × **10** ^−**2**^	0.048	0.022	**2.74** × **10** ^−**2**^
*ABCG8*	rs6544713	0.011	0.018	5.31 × 10^−1^	−0.034	0.014	**1.15** × **10** ^−**2**^	−0.001	0.013	9.49 × 10^−1^	0.005	0.012	6.99 × 10^−1^
*GCKR*	rs1260326	−0.010	0.016	5.22 × 10^−1^	−0.028	0.012	**2.23** × **10** ^−**2**^	0.005	0.013	7.13 × 10^−1^	−0.001	0.011	9.55 × 10^−1^
*RAB3GAP1*	rs7570971	0.003	0.016	8.41 × 10^−1^	0.037	0.013	**4.73** × **10** ^−**3**^	0.023	0.013	6.64 × 10^−2^	0.010	0.011	3.59 × 10^−1^
*DOK7*	rs6831256	0.000	0.015	9.82 × 10^−1^	0.042	0.012	**6.42** × **10** ^−**4**^	0.033	0.012	**8.36** × **10** ^−**3**^	0.011	0.011	2.82 × 10^−1^
*HMGCR*	rs12916	0.024	0.016	1.30 × 10^−1^	−0.036	0.012	**2.73** × **10** ^−**3**^	−0.004	0.012	7.21 × 10^−1^	0.004	0.011	6.81 × 10^−1^
*TIMD4*	rs6882076	−0.012	0.015	4.53 × 10^−1^	0.031	0.012	**1.34** × **10** ^−**2**^	0.028	0.013	2.89 × 10^−2^	0.009	0.010	3.72 × 10^−1^
*FRK*	rs3798236	−0.016	0.017	3.44 × 10^−1^	−0.038	0.013	**3.59** × **10** ^−**3**^	0.002	0.013	8.66 × 10^−1^	−0.001	0.011	9.25 × 10^−1^
*HLA*	rs3177928	0.003	0.022	8.75 × 10^−1^	0.051	0.018	**4.27** × **10** ^−**3**^	0.006	0.018	7.51 × 10^−1^	0.013	0.015	3.93 × 10^−1^
*LPA*	rs1564348	−0.049	0.021	**1.72** × **10** ^−**2**^	0.023	0.017	1.78 × 10^−1^	0.014	0.017	4.15 × 10^−1^	−0.018	0.014	2.04 × 10^−1^
*MIR148A*	rs4722551	−0.076	0.021	**2.50** × **10** ^−**4**^	0.031	0.017	6.92 × 10^−2^	0.024	0.017	1.72 × 10^−1^	−0.024	0.014	9.35 × 10^−2^
*NAT2*	rs4921914	−0.037	0.018	**4.37** × **10** ^−**2**^	−0.030	0.015	3.94 × 10^−2^	−0.021	0.015	1.54 × 10^−1^	−0.023	0.012	6.94 × 10^−2^
*ABCA1*	rs1883025	0.034	0.017	**4.45** × **10** ^−**2**^	0.016	0.014	2.31 × 10^−1^	0.002	0.014	8.68 × 10^−1^	0.015	0.012	2.07 × 10^−1^
*ABO*	rs635634	−0.003	0.019	8.60 × 10^−1^	0.036	0.016	**2.54** × **10** ^−**2**^	0.043	0.015	**5.85** × **10** ^−**3**^	0.014	0.013	2.98 × 10^−1^
*TTC39B*	rs581080	−0.002	0.019	9.06 × 10^−1^	0.039	0.016	**1.20** × **10** ^−**2**^	0.024	0.016	1.24 × 10^−1^	0.007	0.013	5.75 × 10^−1^
*BUD13*	rs12292921	−0.050	0.033	1.30 × 10^−1^	−0.046	0.027	9.08 × 10^−2^	−0.054	0.028	5.79 × 10^−2^	−0.052	0.023	**2.29** × **10** ^−**2**^
*HNF1A*	rs1169288	−0.015	0.017	3.84 × 10^−1^	−0.026	0.013	**4.36** × **10** ^−**2**^	−0.017	0.013	1.79 × 10^−1^	−0.005	0.011	6.70 × 10^−1^
*DLG4*	rs314253	−0.016	0.017	3.38 × 10^−1^	0.036	0.013	**8.47** × **10** ^−**3**^	0.021	0.014	1.28 × 10^−1^	0.012	0.011	2.73 × 10^−1^
*C17orf157*	rs12452315	0.045	0.015	**2.99** × **10** ^−**3**^	0.007	0.013	6.05 × 10^−1^	0.015	0.013	2.48 × 10^−1^	0.026	0.010	**1.41** × **10** ^−**2**^
*LIPG*	rs7240405	0.000	0.021	9.95 × 10^−1^	0.044	0.018	**1.44** × **10** ^−**2**^	0.020	0.018	2.60 × 10^−1^	0.008	0.015	5.98 × 10^−1^
*FUT2*	rs492602	0.005	0.016	7.62 × 10^−1^	0.025	0.013	5.51 × 10^−2^	0.028	0.013	**2.73** × **10** ^−**2**^	0.014	0.011	1.82 × 10^−1^
*LDLR*	rs6511720	0.026	0.023	2.66 × 10^−1^	0.033	0.019	7.54 × 10^−2^	0.028	0.019	1.40 × 10^−1^	0.035	0.016	**2.60** × **10** ^−**2**^
*TOMM40/APOE/APOC1*	rs2075650	0.019	0.021	3.52 × 10^−1^	0.073	0.017	**2.13** × **10** ^−**5**^	0.058	0.016	**5.50** × **10** ^−**4**^	0.042	0.014	**3.29** × **10** ^−**3**^
*FER1LA*	rs2277862	0.043	0.022	5.18 × 10^−2^	0.049	0.018	**5.72** × **10** ^−**3**^	0.047	0.017	**7.30** × **10** ^−**3**^	0.031	0.015	**4.28** × **10** ^−**2**^
*TOP1*	rs6065311	−0.007	0.016	6.42 × 10^−1^	−0.007	0.013	5.90 × 10^−1^	−0.026	0.013	**4.59** × **10** ^−**2**^	−0.012	0.011	2.87 × 10^−1^
*GS*	GS	−0.001	0.002	7.31 × 10^−1^	0.009	0.002	**7.27** × **10** ^−**7**^	0.009	0.002	**4.07** × **10** ^−**6**^	0.004	0.002	**3.78** × **10** ^−**3**^
**HDL**													
*ZNF648*	rs1689800	0.013	0.022	5.63 × 10^−1^	−0.045	0.016	**6.27** × **10** ^−**3**^	−0.001	0.019	9.70 × 10^−1^	0.001	0.014	9.54 × 10^−1^
*APOB*	rs1042034	0.028	0.026	2.85 × 10^−1^	0.042	0.021	**4.04** × **10** ^−**2**^	0.039	0.023	9.01 × 10^−2^	0.026	0.017	1.22 × 10^−1^
*ATG7*	rs2606736	−0.045	0.022	**4.30** × **10** ^−**2**^	0.000	0.017	9.98 × 10^−1^	0.006	0.019	7.61 × 10^−1^	−0.031	0.014	**2.45** × **10** ^−**2**^
*FAM13A*	rs3822072	−0.018	0.021	3.88 × 10^−1^	0.033	0.015	**2.73** × **10** ^−**2**^	0.011	0.017	5.28 × 10^−1^	−0.006	0.013	6.61 × 10^−1^
*ARL15*	rs6450176	0.025	0.024	2.86 × 10^−1^	−0.044	0.018	**1.32** × **10** ^−**2**^	−0.027	0.020	1.78 × 10^−1^	−0.002	0.015	8.92 × 10^−1^
*RSPO3*	rs1936800	0.032	0.022	1.43 × 10^−1^	−0.033	0.015	**3.25** × **10** ^−**2**^	-0.017	0.017	3.20 × 10^−1^	0.015	0.013	2.48 × 10^−1^
*VEGFA*	rs998584	0.025	0.021	2.26 × 10^−1^	−0.012	0.016	4.46 × 10^−1^	0.025	0.018	1.70 × 10^−1^	0.027	0.013	**3.95** × **10** ^−**2**^
*DAGLB*	rs702485	0.010	0.022	6.51 × 10^−1^	0.020	0.017	2.43 × 10^−1^	0.042	0.019	**3.19** × **10** ^−**2**^	0.025	0.014	7.54 × 10^−2^
*IKZF1*	rs4917014	0.031	0.023	1.76 × 10^−1^	−0.039	0.017	**2.14** × **10** ^−**2**^	−0.035	0.019	6.56 × 10^−2^	0.016	0.014	2.56 × 10^−1^
*KLF14*	rs4731702	0.009	0.021	6.62 × 10^−1^	0.031	0.016	**4.68** × **10** ^−**2**^	0.010	0.017	5.83 × 10^−1^	0.003	0.013	8.44 × 10^−1^
*TRIB1*	rs2954029	0.035	0.023	1.25 × 10^−1^	0.036	0.017	**3.32** × **10** ^−**2**^	0.022	0.019	2.45 × 10^−1^	0.013	0.014	3.48 × 10^−1^
*TRPS1*	rs2293889	0.019	0.021	3.70 × 10^−1^	0.038	0.015	**1.29** × **10** ^−**2**^	0.017	0.017	3.13 × 10^−1^	0.009	0.013	5.01 × 10^−1^
*ABCA1*	rs1883025	0.048	0.024	**4.52** × **10** ^−**2**^	0.052	0.018	**3.20** × **10** ^−**3**^	0.046	0.020	**2.02** × **10** ^−**2**^	0.038	0.015	**8.27** × **10** ^−**3**^
*TTC39B*	rs581080	−0.001	0.027	9.63 × 10^−1^	0.045	0.020	**2.51** × **10** ^−**2**^	0.015	0.023	4.97 × 10^−1^	0.008	0.017	6.18 × 10^−1^
*AMPD3*	rs2923084	−0.035	0.027	1.98 × 10^−1^	0.043	0.020	**3.78** × **10** ^−**2**^	0.114	0.024	**1.89** × **10** ^−**6**^	0.015	0.017	3.69 × 10^−1^
*FADS1-2-3*	rs174546	−0.012	0.022	5.99 × 10^−1^	0.049	0.017	**3.44** × **10** ^−**3**^	0.026	0.019	1.69 × 10^−1^	0.017	0.014	2.04 × 10^−1^
*LRP4*	rs3136441	0.003	0.031	9.12 × 10^−1^	−0.048	0.023	**3.71** × **10** ^−**2**^	−0.044	0.027	9.86 × 10^−2^	−0.001	0.020	9.40 × 10^−1^
*MADD/FOLH1*	rs7395662	0.010	0.022	6.52 × 10^−1^	0.051	0.016	**1.87** × **10** ^−**3**^	0.041	0.019	**3.01** × **10** ^−**2**^	0.021	0.014	1.29 × 10^−1^
*MOGAT2*	rs499974	−0.042	0.029	1.49 × 10^−1^	−0.042	0.023	6.13 × 10^−2^	−0.052	0.026	**4.87** × **10** ^−**2**^	−0.032	0.018	8.12 × 10^−2^
*PCNXL3*	rs12801636	−0.028	0.026	2.84 × 10^−1^	−0.036	0.019	6.93 × 10^−2^	−0.053	0.022	**1.73** × **10** ^−**2**^	−0.027	0.016	1.02 × 10^−1^
*LRP1*	rs11613352	−0.069	0.025	**5.91** × **10** ^−**3**^	0.002	0.019	9.06 × 10^−1^	0.009	0.021	6.54 × 10^−1^	−0.029	0.015	6.01 × 10^−2^
*MMAB/MVK*	rs7298565	0.065	0.020	**1.48** × **10** ^−**3**^	−0.015	0.015	3.13 × 10^−1^	−0.011	0.017	5.04 × 10^−1^	0.026	0.013	**4.10** × **10** ^−**2**^
*SCARB1*	rs838880	0.018	0.023	4.50 × 10^−1^	0.008	0.018	6.32 × 10^−1^	0.020	0.020	2.99 × 10^−1^	0.024	0.014	**8.93** × **10** ^−**2**^
*LIPC*	rs1077834	0.058	0.025	**2.34** × **10** ^−**2**^	0.072	0.019	**2.32** × **10** ^−**4**^	0.029	0.023	2.07 × 10^−1^	0.045	0.016	**4.99** × **10** ^−**3**^
*CETP*	rs3764261	0.047	0.023	**4.12** × **10** ^−**2**^	0.055	0.017	**1.65** × **10** ^−**3**^	0.068	0.020	**7.62** × **10** ^−**4**^	0.064	0.014	**7.11** × **10** ^−**6**^
*CMIP*	rs2925979	0.019	0.024	4.33 × 10^−1^	−0.062	0.018	**5.31** × **10** ^−**4**^	−0.032	0.020	1.14 × 10^−1^	−0.012	0.015	4.36 × 10^−1^
*LCAT*	rs16942887	0.015	0.031	6.32 × 10^−1^	0.048	0.024	**4.55** × **10** ^−**2**^	0.020	0.028	4.81 × 10^−1^	0.017	0.020	3.84 × 10^−1^
*ABCA8*	rs4148008	−0.035	0.022	1.12 × 10^−1^	−0.047	0.017	**5.73** × **10** ^−**3**^	−0.035	0.019	6.83 × 10^−2^	−0.039	0.014	**4.64** × **10** ^−**3**^
*PGS1*	rs4129767	0.064	0.022	**3.54** × **10** ^−**3**^	−0.036	0.016	**2.61** × **10** ^−**2**^	−0.027	0.018	1.32 × 10^−1^	0.021	0.013	1.26 × 10^−1^
*PLTP*	rs6065906	0.001	0.027	9.67 × 10^−1^	0.054	0.021	**8.98** × **10** ^−**3**^	0.053	0.023	**2.39** × **10** ^−**2**^	0.017	0.017	3.09 × 10^−1^
*GS*	GS	0.006	0.003	**2.15** × **10** ^−**2**^	0.003	0.002	1.59 × 10^−1^	0.004	0.002	1.26 × 10^−1^	0.005	0.002	**3.27** × **10** ^−**3**^
**TRIGLYCERIDES**													
*APOB*	rs1042034	−0.025	0.044	5.73 × 10^−1^	0.069	0.033	**3.72** × **10** ^−**2**^	0.000	0.038	9.95 × 10^−1^	0.011	0.026	6.79 × 10^−1^
*GCKR*	rs1260326	0.056	0.036	1.28 × 10^−1^	0.098	0.025	**8.72** × **10** ^−**5**^	0.025	0.030	3.98 × 10^−1^	0.047	0.021	**2.61** × **10** ^−**2**^
*IRS1*	rs2972146	−0.086	0.039	**2.92** × **10** ^−**2**^	−0.010	0.029	7.41 × 10^−1^	−0.020	0.034	5.56 × 10^−1^	−0.037	0.023	1.10 × 10^−1^
*MSL2L1*	rs645040	−0.003	0.041	9.48 × 10^−1^	0.079	0.030	**9.26** × **10** ^−**3**^	−0.021	0.036	5.70 × 10^−1^	0.010	0.024	6.90 × 10^−1^
*MAP3K1*	rs9686661	0.004	0.043	9.26 × 10^−1^	0.090	0.031	**3.31** × **10** ^−**3**^	0.045	0.037	2.23 × 10^−1^	0.029	0.025	2.40 × 10^−1^
*MLXIPL*	rs17145738	−0.011	0.056	8.49 × 10^−1^	0.082	0.039	**3.79** × **10** ^−**2**^	−0.084	0.046	6.83 × 10^−2^	−0.029	0.033	3.72 × 10^−1^
*TRIB1*	rs2954029	0.037	0.038	3.30 × 10^−1^	0.074	0.027	**5.94** × **10** ^−**3**^	−0.025	0.031	4.23 × 10^−1^	0.015	0.022	4.92 × 10^−1^
*BUD13*	rs12292921	0.129	0.076	9.07 × 10^−2^	−0.057	0.056	3.17 × 10^−1^	−0.145	0.067	**3.16** × **10** ^−**2**^	−0.019	0.045	6.80 × × 10^−1^
*FADS1-2-3*	rs174546	−0.032	0.036	3.77 × 10^−1^	0.077	0.027	**4.02** × **10** ^−**3**^	0.054	0.032	8.98 × 10^−2^	0.016	0.021	4.50 × 10^−1^
*LRP1*	rs11613352	−0.043	0.041	2.99 × 10^−1^	0.009	0.030	7.58 × 10^−1^	0.079	0.035	**2.39** × **10** ^−**2**^	0.005	0.024	8.36 × 10^−1^
*ZNF664*	rs11057408	−0.066	0.038	8.33 × 10^−2^	−0.062	0.027	**2.34** × **10** ^−**2**^	−0.020	0.032	5.24 × 10^−1^	−0.033	0.022	1.32 × 10^−1^
*APOC2/APOCL/APOE*	rs439401	−0.043	0.036	2.34 × 10^−1^	−0.017	0.025	4.96 × 10^−1^	0.076	0.029	**8.92** × **10** ^−**3**^	0.016	0.021	4.37 × 10^−1^
*CILP2*	rs10401969	−0.090	0.072	2.09 × 10^−1^	0.146	0.050	3.40 × 10^−3^	0.199	0.063	**1.64** × **10** ^−**3**^	0.047	0.041	2.52 × 10^−1^
*INSR*	rs7248104	0.028	0.036	4.44 × 10^−1^	0.017	0.025	4.98 × 10^−1^	0.072	0.029	**1.39** × **10** ^−**2**^	0.057	0.021	**6.65** × **10** ^−**3**^
*PLTP*	rs6065906	0.016	0.045	7.16 × 10^−1^	0.069	0.033	**3.91** × **10** ^−**2**^	0.053	0.039	1.72 × 10^−1^	0.022	0.027	4.11 × 10^−1^
*GS*	GS	0.000	0.007	9.86 × 10^−1^	0.010	0.005	**4.37** × **10** ^−**2**^	0.014	0.006	**1.06** × **10** ^−**2**^	0.008	0.004	**3.08** × **10** ^−**2**^

Beta and SE at birth, 3 year and 5 year are natural logarithm of the lipid level trait in mg/dl. The unit of beta and SE after linear mixed model is natural logarithm of the lipid level trait in mg/dl per year.

### Association between child SNPs/GS and lipid levels

The GS had a statistically significant and directionally consistent association with LDL-C at 3 and 5 years of age and from birth to 5 years of age by using the linear mixed-effect model (β = 0.017 ± 0.003, *P* = 2.9 × 10^−8^, β = 0.020 ± 0.003, *P* = 5.7 × 10^−9^ and β = 0.013 ± 0.003, *P* = 2.6 × 10^−7^ respectively) (Table 2 and Supplementary Table 3). The GS also had a statistically significant and directionally consistent association with TC at 3 and 5 years of age (β = 0.009 ± 0.002, *P* = 9.1 × 10^−7^ and β = 0.009 ± 0.002, *P* = 7.7 × 10^−6^ respectively) (Table 2 and Supplementary Table 4). A statistically significant and directionally consistent association was observed between *AMPD3* rs2923084 and HDL-C at 5 years of age (β = 0.096 ± 0.024, *P* = 9.7 × 10^−5^) (Table 3 and Supplementary Table 5). A statistically significant and directionally consistent association between *CETP* rs3764261 and HDL-C was also observed from birth to 5 years of age by using the mixed effect model (β = 0.064 ± 0.014, *P* =  7.4 × 10^−6^) (Table 3 and Supplementary Table 5).

In addition to these association, nominal consistent associations were found for at least 2 time measurements with 1) TC for rs4921914 (*NAT2*), rs6756629 (*ABCG5*)*,* rs6882076 (*TIMD4*), rs635634 (*ABO*), rs2075650 (*TOMM40*) and rs2277862 (*FER1LA*), 2) LDL-C for rs6756629 (*ABCG5*), rs6882076 (*TIMD4*), rs2327951 (*MYLIP/GMPR*), rs635634 (*ABO*) and rs8017377 (*NYNRIN*), 3) HDL-C for rs1883025 (*ABCA1*), rs1077834 (*LIPC*), rs3764261 (*CETP*), rs4917014 (*IKZF1*), rs2923084 (*AMPD3*), rs7395662 (*MADD*) and rs6065906 (*PLTP*) and 4) TG for rs10401969 (*CILP2*).

### Age-dependent genetic effect in children

We compared the beta values across the different times of measurement (i.e. birth, 3 years and 5 years of age) for the SNPs/GS that presented statistically significant associations with lipid traits (Supplementary Table 7). The TC GS showed an increase of its beta values between birth, 3 years and 5 years of age (*P*_*0-3*_ = 5.9 × 10^−4^ and *P*_*0-5*_ = 9.6 × 10^−4^, respectively). The LDL-C GS also showed an increase of its beta values between birth and 3 years of age and also between birth and 5 years of age (*P*_*0-3*_ = 1.9 × 10^−2^ and *P*_*0-5*_ = 4.1 × 10^−3^, respectively). On the contrary, the *CETP* rs3764261 SNP did not show any significant increase of its beta-values between birth, 3 and 5 years of age (*P*_*0-3*_ = 0.31, *P*_*3-5*_ = 0.34, *P*_*0-5*_ = 0.21, respectively). The AMPD3 rs2923084 SNP effect’s between birth and 3 year of age was not statistically significant (*P*_*0-3*_ = 6.50 × 10^−2^) whereas significant differences were observed between year 3 and 5 and at birth and 5 years (*P*_*0-3*_ = 2.30 × 10^−2^ and *P*_*0-5*_ = 3.71 × 10^−5^, respectively).

### Comparison of the genetic effects in child and adult populations

We compared the beta values obtained in children using linear mixed-effect models and those obtained by the Global Lipids Genetics Consortium in adults (Table 4). Of the six SNPs nominally associated with TC in children, adult beta values were significantly higher for rs12292921 (*APOA1*) and children beta values were higher for rs12452315 (*OSBPL7*) and rs2277862 (*FER1LA*). In regards to the two SNPs associated with TG only, rs7248104 (*INSR*) showed a significantly larger effect in adults in comparison with children (Table 4). With respect to HDL-C, two SNPs (rs2606736 (*ATG7*) and rs4148008 (*ABCA8*)) out of the six showed a significant difference in their beta values, the effects being smaller in children when compared to adults. Lastly, rs12292921 (*APOA1*) and rs12452315 (*OSBPL7*) out of the eight SNPs nominally associated with LDL-C showed a significantly smaller and higher effect, respectively, in adults from the Global Lipids Genetics Consortium in comparison to children from FAMILY.

**Table 4:** Comparison of the statistically nominal significant SNP effects between children from FAMILY and adults from the Global Lipids Genetics Consortium.

Trait	Gene	SNP	Consortium			FAMILY			Z-test	p-value
			Beta	SE	p-value	Beta	SE	p-value		
TC	*ABCG5*	rs6756629	0.122	0.010	4.35 × 10^−36^	0.219	0.100	2.88 × 10^−2^	−0.965	1.67 × 10^−1^
TC	*APOA1/APOA4/APOA5/APOC3*	rs12292921	0.096	0.007	2.22 × 10^−42^	−0.193	0.107	6.99 × 10^−2^	2.695	**3.52** × **10** ^−**3**^
TC	*OSBPL7*	rs12452315	0.024	0.004	2.67 × 10^−10^	0.123	0.048	1.01 × 10^−2^	−2.055	**1.99** × **10** ^−**2**^
TC	*LDLR*	rs6511720	0.185	0.006	5.43 × 10^−202^	0.188	0.073	1.02 × 10^−2^	−0.041	4.84 × 10^−1^
TC	*TOMM40/APOE/APOC1*	rs2075650	0.143	0.005	8.93 × 10^−158^	0.230	0.065	3.91 × 10^−4^	−1.335	9.10 × 10^−2^
TC	*FER1LA*	rs2277862	0.035	0.005	2.26 × 10^−13^	0.154	0.069	2.54 × 10^−2^	−1.720	**4.27** × **10** ^−**2**^
HDL	*ATG7*	rs2606736	0.025	0.004	4.80 × 10^−8^	−0.098	0.050	5.23 × 10^−2^	2.452	**7.10** × **10** ^−**3**^
HDL	*VEGFA*	rs998584	0.026	0.004	2.27 × 10^−11^	0.094	0.048	4.87 × 10^−2^	−1.412	7.90 × 10^−2^
HDL	*ABCA1*	rs1883025				0.143	0.053	7.53 × 10^−3^		
HDL	*LIPC*	rs1077834	0.125	0.004	7.77 × 10^−180^	0.171	0.058	3.28 × 10^−3^	−0.791	2.14 × 10^−1^
HDL	*CETP*	rs3764261	0.241	0.004	1.39 × 10^−769^	0.238	0.052	5.38 × 10^−6^	0.058	4.77 × 10^−1^
HDL	*ABCA8*	rs4148008	0.028	0.004	1.13 × 10^−12^	−0.139	0.051	5.85 × 10^−3^	3.264	**5.48** × **10** ^−**4**^
LDL	*CELSR2/PSRC1/SORT1*	rs646776	0.160	0.004	1.63 × 10^−272^	0.158	0.061	9.83 × 10^−3^	0.033	4.87 × 10^−1^
LDL	*ABCG5*	rs6756629	0.129	0.010	1.77 × 10^−34^	0.213	0.103	3.92 × 10^−2^	−0.812	2.08 × 10^−1^
LDL	*APOB*	rs1367117	0.119	0.004	9.48 × 10^−183^	0.134	0.053	1.20 × 10^−2^	−0.282	3.89 × 10^−1^
LDL	*APOA1/APOA4/APOA5/APOC3*	rs12292921	0.068	0.007	7.21 × 10^−20^	−0.175	0.110	1.11 × 10^−1^	2.205	**1.37** × **10** ^−**2**^
LDL	*BRCA2*	rs4942486	0.024	0.004	2.26 × 10^−11^	0.082	0.050	9.85 × 10^−2^	−1.156	1.24 × 10^−1^
LDL	*OSBPL7*	rs12452315	0.025	0.004	2.37 × 10^−10^	0.114	0.049	2.17 × 10^−2^	−1.810	**3.51** × **10** ^−**2**^
LDL	*LDLR*	rs6511720	0.221	0.006	3.85 × 10^−262^	0.194	0.075	1.02 × 10^−2^	0.359	3.60 × 10^−1^
LDL	*TOMM40/APOE/APOC1*	rs157580	0.108	0.005	9.24 × 10^−119^	0.128	0.051	1.22 × 10^−2^	−0.390	3.48 × 10^−1^
TG	*GCKR*	rs1260326	0.115	0.003	2.29 × 10^−239^	0.116	0.048	1.28 × 10^−2^	−0.021	4.92 × 10^−1^
TG	*INSR*	rs7248104	0.022	0.003	5.05 × 10^−10^	0.138	0.047	3.28 × 10^−3^	−2.463	**6.89** × **10** ^−**3**^

*The P-Values are for each child and adult SNP to show association effect in adults from the Global Lipids Genetics Consortium in comparison to children from FAMILY study. Beta and SE at birth, 3 year and 5 year are quartile normalization of the lipid level trait in mg/dl.

### Comparison of the variance explained by the 158 SNPs in child and adult populations

We computed the variances explained by the SNPs used in the GS at 3 times of measurements for the four traits and we compared our variances with the theoretical variance calculated from the summary statistics of the the Global Lipids Genetics Consortium data in adults^8^. Results are available in Table 5. In adult populations, we found that the 69 SNPs associated with TC explained 6.4% of the theoretical variance, 7.1% for the 73 SNPs associated with HDL-C, 7.4% for the 59 SNPs associated with LDL-C and 4.2% for the 40 SNPs associated with TG. Child SNPs explained almost all the adult variance for TC and LDL at year 3 and year 5 whereas SNPs associated with HDL and triglycerides explained only a small part of the adult variance. At birth, SNPs associated with all the lipid traits explained only a negligible part of the adult variance.

**Table 5:** Percentage of the variance explained in children by the lipids level associated SNPs at each time of measurement for the different traits. Variances in adults were computed from summary statistic of the consortium data (Willer *et al*. Nat. Genet 2013).

Years	Adults	Birth	3 years	5 years
TC (69 SNPs)	6.4	0.56	6.74	4.14
LDL-C (59 SNPs)	7.4	0.17	5.18	5.07
HDL-C (73 SNPs)	7.1	0.55	0.99	0.51
TG (40 SNPs)	4.2	0.00	0.55	0.55

## Discussion

We explored the associations of 158 SNPs detected in adults that reached genome-wide statistically significant level of association (*P* < 5 × 10^−8^) with HDL-C, LDL-C, TC, and TG levels in a population with predominantly European ancestry from the FAMILY birth cohort. This is the first report to study lipid SNPs associated to adults in an early child longitudinal birth cohort. We found a statistically significant association of GS with LDL-C levels at 3 and 5 years of age and from birth to 5 years of age by using a mixed effect model. We also found an association of GS with TC levels at 3 and 5 years of age. A statistically significant association was identified between *AMPD3* rs2923084 and HDL-C level at 5 years of age, as well as an association of *CETP* rs3764261 with HDL-C from birth to 5 years of age when using the mixed-effect model. The AMP enzyme coded by *AMPD3* interacts with lipids in cytosolic regulation process^13^. CETP acts in cholesterol transport as a central effector between HDL-C and apolipoprotein B^14, 15^. This role makes CETP a relevant molecular target for novel lipid modifying drugs^15^. Interestingly, the effect size of several SNPs and the GS are different in diverse age windows (i.e. values at birth compared to those at 5 years of age), suggesting there is an overall variable effect of genetic factors on blood lipid levels in early life. The nominal evidence of association observed for the GS of HDL-C and TG can be explained by the modest power of our study and differential heritability of lipid levels in early childhood. Our data are consistent with reports that demonstrate an increase in heritability values of lipid traits across adolescence^16^. Hypothesis-generating GWAS for lipid traits in younger populations have yet to be reported. Applying these approaches in diverse ethnic populations may shed further light on the genetic architecture of lipids.

### Strengths and Limitations

Our study has several strengths. This is the first study to examine SNPs that affect lipid levels at birth and early childhood. Secondly, we presented an exhaustive list of lipid associated SNPs (*n* = 158) extracted from the Illumina Cardio-metabochip. Lastly, the familial-based design also provided excellent quality control for genotyping data by assessing Mendelian inconsistencies.

The limitations of this study include the modest size of the sample that may have decreased the power to detect associations with small effect sizes and/or low risk allele frequencies (Supplementary Figures 1–4). The power of our study was computed to be 80% using QUANTO software. All the range of allele frequencies (5% to 95%) and 4 different beta effects were assessed. In our study, we have the power to detect beta effect upper of 0.08 mg/dl for allele frequencies from 10% to 90%. Another limitation of this study is the lack of the replication, but there are only few studies using children in this age range with lipid level measurements and genetic data. The follow-up duration in this report (birth to 5 years of age) prevents the assessment of genetic effects of the lipid-associated SNPs later on in childhood and adolescence. It should be noted that since FAMILY is an ongoing longitudinal cohort, an opportunity to reanalyze the data at a later stage of life is possible. The availability of atherosclerosis measurements at 10-years of age through intima media thickness makes this perspective even more relevant. Lastly, we used cord blood lipid measurements at birth since fasting samples from newborns are not available. Use of cord blood may have biased our interpretation of the data since the phenotype is not defined in the same way at all-time points.

## Conclusion

In conclusion, we demonstrate that 158 loci known to be associated with blood lipids in adults are also associated with TC, LDL-C and HDL-C amongst children from birth to 5 years of age. Our results suggest that genes predisposing to abnormal lipid levels in adults already have an impact during the first years of life. The discovery that genetic variants modulate LDL-C early in life is striking as high LDL-C levels have been shown to be causally associated with future coronary artery disease events^17^. This has some importance in public health especially in the context of earlier stages of CVD prevention strategies in order to lower blood lipid levels in genetically at risk subgroups. Those with a genetic risk for CVD may be identified through a family history of dyslipidemia or through individualized genetic medicine approaches, once an exhaustive list of lipid associated genetic variants is completed. The practice of administrating lipid lowering drugs restricted to adults may be challenged as our findings suggest that childhood preventative strategies would be beneficial in order to prevent CVD more efficiently.

## Methods

### Study population

The details of the FAMILY study have been described in a previous publication^18^. FAMILY is an ongoing birth cohort study that includes mothers, fathers and children with a planned follow-up of 10 years. Over the last 7 years, 859 families (901 babies, 259 siblings, 857 mothers and 530 fathers) have been enrolled into the FAMILY study and followed longitudinally. In this study, we excluded offspring from multiple births (as the twin status has a strong impact on birth weight and postnatal catch-up) and siblings of “index” children due to familial relatedness and phenotypic issues (i.e. absence of phenotypic data at birth). Following these exclusion criteria, 544 mothers, 352 fathers and 544 children had DNA extracted and were successfully genotyped. Phenotypic characteristics of these individuals are displayed in Table 1. Genetic and clinical data from the FAMILY study were centralized and coordinated at the Population Health Research Institute (Hamilton, ON, Canada). The study was approved by the Research Ethics Boards at the participating hospitals: Hamilton Health Sciences, St Joseph’s Hospital – Hamilton, ON, Canada and Joseph Brant Memorial Hospital, Burlington, ON, Canada. Written informed consent was obtained from all the adult participants and the parent provided consent for their children prior to participation, in accordance with the Declaration of Helsinki. All experiments were performed in accordance with relevant guidelines and regulations of McMaster University.

### Phenotyping

Offspring’s phenotypic measurements have been performed at birth, 3 years and 5 years of age. Umbilical venous cord blood for analyses of the newborn’s lipids was collected in the Labour and Delivery unit immediately following birth. Fasting blood samples were taken from the children at the third and fifth year visits for measurement of lipid levels, at the Clinical Trials Clinical Research Laboratory (CTCRL) Hamilton Health Sciences. The blood samples were initially stored at 4 °C, processed within 15 hours and stored long term at −165 °C in liquid nitrogen. TC, HDL-C and TG were measured using enzymatic methods on the ROCHE INTEGRA analyzer and LDL-C was calculated using the Friedewald equation^19^. This formula has been validated at birth and kids accordingly to Hardell *et al.* ^20^ and Yu *et al.* ^21^.

### Genotyping

Genomic DNAs were extracted from buffy coats for all the participants. The genotyping was performed using the Illumina Cardio-Metabochip (San Diego, CA, USA). This array has been designed by seven consortia studying cardiac, metabolic and anthropometric traits. A selection of 196,725 SNPs for 23 different traits was made. The design and SNP selection of the array are detailed elsewhere^22^. We selected SNPs that reached genome-wide statistically significant level of association (*P* < 5 × 10^−8^) for TC, LDL-C, HDL-C or TG in at least one population of European ancestry that were available in the Cardio-Metabochip array. An independent search by S.C. and S.R.d.P. allowed the extraction of the lipid-associated SNPs from two databases (HuGE Navigator^23^ and NHGRI GWAS Catalog^24^) or by a manual literature search in the Pubmed database using the following key words: “Genome wide association study”, “lipoprotein cholesterol”, “high-density lipoprotein cholesterol”, “total cholesterol” and “triglycerides”. For SNPs that were not available in the Cardio-Metabochip, we identified proxy SNPs using the Broad Institute website tool SNAP (SNP Annotation and Proxy Search) as well as the Cardio-Metabochip file provided by Illumina using chromosome and chromosomal positions of SNPs from the NCBI Human Genome Browser^25, 26^. We used the following criteria to select proxy SNPs: 1) SNPs included in the Cardio-Metabochip 2) r^2^ > 0.95 in European population data issued from the 1000 Genomes Project^27^, 3) selection of a coding non-synonymous SNP if available in the proxy list, otherwise, the SNP located closest to the GWAS lead SNP was selected. Linkage disequilibrium (LD) between the selected SNPs was evaluated by using SNAP^25^ in European population data of the 1000 Genomes Project^27^. We discarded 96 SNPs that displayed r^2^ > 0.2 with another SNP in the list. In total, we selected 158 lipid-associated polymorphisms (Supplementary Table 1 and Supplementary Figure 5). Standard quality control was used to assess the quality of the genotyping. Twenty-six individuals who displayed SNP missing rates >10% for the Metabochip were discarded. All 158 SNPs displayed call rates >97% and obeyed to Hardy Weinberg Equilibrium (P ≥1 × 10^−6^, Supplementary Table 2). As an additional quality control procedure, we analyzed the Mendelian transmission patterns of the 158 SNPs. We found recurrent Mendelian inconsistencies in 5 pedigrees. After excluding the 5 non-biological fathers from the analysis, only two Mendelian distortions was observed in the sample of 158 SNPs, which therefore successfully passed the quality control test. We also tested the self-reported ethnicity using principal component analysis EIGENSTRAT^28^. We found that 92.8% of mothers, 89.3% of fathers and 91.1% of children had European ancestry.

### Statistical analyses

We coded genotypes as 0, 1 and 2, depending on the number of copies of the lipid increasing alleles. Four genotype scores (GS) were calculated by summing the alleles of 69, 59, 73, 40 SNPs for TC, LDL-C, HDL-C and TG, respectively. We used a unweighted GS as recommended by previous studies^29, 30^. Missing values were imputed using the method of the mean in the calculation of the GS. This imputation was performed for each SNP individually using the arithmetic average of the coded genotypes observed for all the successfully genotyped individuals.

Associations between child SNP/GS and lipid measurements were assessed using linear regression at each time of measurement. Our model was adjusted for sex, age and the 10 first principal components. The linear model can be calculated using: ln(Lipid level) ~SNP + sex + age + PCAs (+parental SNP) + residuals.

The linear mixed-effect regression model was utilized to account for the longitudinal nature of the data (3 lipid measurements across the follow up). In addition, the results of the linear mixed model allow an assessment of the association between SNP/GS across the follow up period. For each trait, the unit of the beta effect and standard deviation are the natural logarithm of the lipid level trait in mg/dl per year and it could be equivalent to a mean variation of level of trait across all the follow-up. We used the intercept as random effects for the linear mixed-effect regression model and sex, age and the 10 first principal components of EIGENSTRAT analysis as fixed effects^28^. The linear mixed model can be simplified to: ln (Lipid level) ~SNP + se + PCAs (+parental SNP) + residual + age + (1|ID). In both models, we used the 10 first principal components as covariates to account for population structure.

Due to their skewedness distribution, the traits were transformed using a natural logarithm (Supplementary Figure 6). All the regression analyses were performed using the free software R 3.0.1. using the packages “lme4” for the linear mixed-effect model.

The Hardy-Weinberg equilibrium was tested using a Chi-square test in combination with permutations and bootstrapping. Mendelian incompatibilities were checked using PLINK. Two-tailed p-values are presented in this manuscript. TC, LDL-C, HDL-C and TG measurements at different times are highly correlated with each other. Similarly, the different statistical tests (linear regression and mixed-model regression) performed in this study are not independent from each other. We therefore only accounted for the number of genetic markers (*n* = 158) and the number of measurements (*n* = 3 at birth, 3 and 5 years) while applying Bonferroni’s correction for multiple testing. We acknowledged the over-conservative nature of the Bonferroni test and the strong prior evidence of association of these SNPs with lipid levels, based on previous GWAS in adult populations. *P* < 1.05 × 10^−4^ (0.05/(158 × 3) was therefore considered as statistically significant. The effect sizes for offspring SNPs were compared at birth, 3 years and 5 years of age using a Z-test. To compare with the child SNPs effect size with the adults we performed a quartile normalization as performed in Willer *et al*.^8^. Variances in our dataset were computed using GCTA using the same covariates as linear models^31^. Theoretical variance from the consortium summary data were compute using the following formula: β_1_^2^ × Var (SNP_1_) + β_2_^2^ × Var (SNP_2_) + …, where β is the SNP effect and Var (SNP) is the genotypic variance (2 × MAF × (1 − MAF)).

## Electronic supplementary material

Supplementary Information
