# Supplementary material for: Genetic contribution to lipid levels in early life based on 158 loci validated in adults: the FAMILY study
Source: Sci Rep. 2017 Mar 6;7:68. doi: 10.1038/s41598-017-00102-1 (PMC5427872; doi:10.1038/s41598-017-00102-1)
Supplement: Supplementary file 1 — Supplementary Information [file 41598_2017_102_MOESM1_ESM.pdf]

SUPPLEMENTARY INFORMATION

Child genetic contribution to lipid levels in early life based on 158 loci validated in adults: the FAMILY study

Shanice Christie[1,#], Sébastien Robiou-du-Pont[1,#], Sonia S. Anand[1,2], Katherine M. Morrison[3], Sarah D. McDonald[1,4], Guillaume Paré[1,5], Stephanie A. Atkinson[3], Koon K. Teo[1,2], David Meyre[1,5,*]

[1]Department of Clinical Epidemiology and Biostatistics, McMaster University, Hamilton, Ontario, Canada; [2]Department of Medicine, McMaster University, Hamilton, Ontario, Canada; [3]Department of Pediatrics, Hamilton Health Sciences and McMaster University, Hamilton, Ontario, Canada; [4]Department of Obstetrics and Gynecology, McMaster University, Hamilton, Ontario, Canada; [5]Department of Pathology and Molecular Medicine, McMaster University, Hamilton, Ontario, Canada.

[*] Corresponding author: David Meyre, Department of Clinical Epidemiology and Biostatistics McMaster University, Michael DeGroote Centre for Learning & Discovery, Room 3205, 1280 Main Street West, Hamilton, ON L8S 4K1, Canada. Tel: 905.525.9140 Ext. 26802; Fax: 905.528.2814; Email:meyred@mcmaster.ca

# These authors contributed equally to the work.

Supplementary Table 1. SNP selection

GWAS SNP	Proxy	R2	CHR	Position in hg18	Gene	Major allele	Minor allele	MAF	Risk allele	Traits and publications
rs4846914			1	8973848	*GALNT2*	A	G	0.401	A/G	TG (2,4), HDL (2,4,6)
rs1077514			1	23638820	*ASAP3*	A	G	0.149	A	TC (2)
rs12027135			1	25648320	*LDLRAP1*	A	T	0.451	T/T	TC / LDL (2,4)
rs4660293			1	25958351	*PABPC4*	A	G	0.229	A	HDL (2,4)
rs12748152	rs12746810	1	1	26885720	*PIGV*	G	A	0.074	G/A/A	HDL/ LDL/TG (2)
rs514230	rs558971	0.97	1	36875979	*IRF2BP2*	G	A	0.464	G/G	TC/LDL (1,2,4)
rs4650994			1	39800767	*ANGPTL1/C1ORF220*	A	G	0.476	G	HDL (2)
rs11206510			1	50087106	*PCSK9*	A	G	0.175	A	LDL (2,6-8)
rs646776			1	53333782	*CELSR2*	A	G	0.221	A/A	TC /LDL (1,4)
rs2479409			1	55277238	*PCSK9*	A	G	0.354	G/G	TC / LDL (2,4)
rs2131925			1	62798530	*ANGPTL3*	C	A	0.337	A/A/A	TC / LDL / TG (2,4)
rs7515577	rs6603981	1	1	135554376	*EVI5*	C	A	0.212		TC (2,4)
rs267733			1	149225460	*ANXA9-CERS2*			0.147		LDL (2)
rs12145743			1	154967275	*RRNAD1*	A	C	0.339	C	HDL (2)
rs1689800			1	180435508	*ZNF648*	A	G	0.368	A	HDL (2,4)
rs2642442	rs2807834	0.96	1	219037216	*MOSC1*	C	A	0.301	A/A	TC / LDL (2,4)
rs515135			2	16303287	*APOB*	G	A	0.190	G	LDL (7)
rs1042034			2	21078786	*APOB*	A	G	0.209	G/A	HDL / TG (4)
rs1367117			2	21117405	*APOB*	G	A	0.310	A/A	TC / LDL (2,4)
rs6756629			2	21139562	*ABCG5*	G	A	0.064	G/G	TC (4)/ LDL (1,4)
rs1260326			2	27584444	*GCKR*	G	A	0.425	A/A	TC (2,4), TG (2,4,6)
rs7570971			2	32508014	*RAB3GAP1*	C	A	0.396	A	TC (2,4)
rs2710642			2	63003061	*EHBP1*	A	G	0.322	A	LDL (2)
rs6544713			2	88249285	*ABCG8*	G	A	0.316	A/A	TC (2), LDL (2,5)
rs10490626			2	118552311	*INSIG2*	G	A	0.080	A/A	LDL/TC (2)
rs2030746			2	121025958	*LOC84931*	G	A	0.391	A/A	LDL/TC (2)
rs10195252			2	161009807	*COBLL1*	A	G	0.403	A	TG (1)
rs12328675			2	165249046	*COBLL1*	A	G	0.126	G	HDL (2,4)
rs2287623			2	169538401	*ABCB11*	A	G	0.405	G	TC (2)
rs11694172			2	203240549	*FAM117B*	A	G	0.236	G	TC (2)
rs1047891	rs7422339	1	2	211540507	*CPS1*	C	A	0.305	C	HDL/ TG (2,4)
rs1250229			2	216012629	*FN1*	G	A	0.264	G	LDL (2)
rs2972146			2	226808942	*IRS1*	A	C	0.355	C/A	HDL / TG (2,4)
rs11563251			2	234344123	*UGT1A1*	G	A	0.112	A/A	TC/LDL (2)
rs6805251			3	3442937	*GSK3B*	G	A	0.396	A	HDL (2)
rs645040			3	11063306	*MSL2L1*	A	C	0.213	A	TG (2,4)
rs2606736			3	11375249	*ATG7*	A	G	0.386	G	HDL (2)
rs2013208			3	50104403	*RBM5*	G	A	0.481	A	HDL (2)
rs13326165			3	52507158	*STAB1*	G	A	0.212	A	HDL (2)
rs7640978			3	122027585	*CMTM6*	G	A	0.073	G/G	LDL/TC (2)
rs17404153			3	133645890	*DNAJC13*	C	A	0.113	C/C	LDL/HDL (2)
rs10019888			4	25672088	*C4orf52**	A	G	0.162	A	HDL (2)

GWAS SNP	Proxy	R2	CHR	Position in hg18	Gene	Major allele	Minor allele	MAF	Risk allele	Traits and publications
rs3822072			4	89960292	*FAM13A*	G	A	0.473	G	HDL (2)
rs2602836			4	100233828	*ADH5*	G	A	0.433	A	HDL (2)
rs13107325			4	103407732	*SLC39A8*	G	A	0.070	G	HDL (2,4)
rs442177			4	122883315	*KLHL8*	A	C	0.421	A	TG (2,4)
rs6831256			4	156322875	*DOK7*	A	G	0.434	G/G/G	TG/ LDL/TC (2)
rs6882076			5	6415797	*TIMD4*	G	A	0.368	G/G/G	TC / LDL / TG (2,4)
rs9686661			5	45333283	*MAP3K1*	G	A	0.207	A	TG (2,4)
rs12916			5	74692295	*HMGCR*	A	G	0.397	G/G	TC (2,4), LDL (1,2,4,7)
rs6450176			5	137409312	*ARL15*	G	A	0.252	G	HDL (2,4)
rs4530754			5	176781935	*CSNK1G3*	A	G	0.453	A/A	LDL/TC (2)
rs998584			6	9220768	*VEGFA*	C	A	0.465	C/A	TG/HDL (2)
rs3757354			6	16235386	*IDOL*	G	A	0.209	G/G	LDL/TC (1)
rs2142672	rs2327951	1	6	16303287	*MYLIP/GMPR*	A	G	0.272	G	LDL (6)
rs1800562			6	26201120	*HFE*	G	A	0.059	G/G	TC / LDL (2,4)
rs3177928			6	32520413	*HLA*	G	A	0.157	A/A	TC/LDL (2,4)
rs2814944			6	34660775	*C6orf106*	G	A	0.160	G	TC (2,4)
rs2758886			6	39358815	*KCNK17*	G	A	0.316	A	TC (2)
rs605066	rs634869	1	6	43987422	*CITED2*	G	A	0.426	G	HDL (2,4)
rs1084651			6	55268627	*LPA*	G	A	0.158	G	HDL (2,4)
rs9488822	rs3798236	1	6	116127173	*FRK*	A	G	0.359	G/G	TC / LDL (2,4)
rs1936800			6	127477757	*RSPO3*	A	G	0.485	G/A	HDL/TG (2)
rs9376090			6	135144821	*HBS1L*	A	G	0.258	A	TC (2)
rs1564348			6	160498850	*LPA*	A	G	0.167	G/G	TC / LDL (4)
rs1997243			7	1050303	*GPR146*	A	G	0.139	G	TC (2)
rs4142995			7	17885783	*SNX13*	C	A	0.395	C	HDL (2)
rs702485			7	20365025	*DAGLB*	A	G	0.449	G	HDL (2)
rs12670798			7	21573877	*DNAH11*	A	G	0.239	G/G	TC (2,4,5), LDL (1,2,4,5)
rs2072183			7	44545705	*NPC1L1*	C	G	0.235	G/G	TC/LDL (2,4)
rs4917014			7	53898229	*IKZF1*	A	C	0.316	C	HDL (2)
rs17145738			7	72620810	*MLXIPL*	G	A	0.130	A.A	TG (1,2,4,8)
rs38855			7	116145280	*MET*	A	G	0.463	A	TG (2)
rs4731702			7	122341080	*KLF14*	G	A	0.480	A	HDL (2,4)
rs4722551			7	130083924	*MIR148A*	A	G	0.159	G/G/G	LDL/TC/TG (2)
rs17173637			7	150160382	*ABP1*	A	G	0.083	A	HDL (2)
rs1832007	rs6601924	1	8	5237302	*AKR1C4*	A	G	0.221	A	HDL (4), TG (2)
rs11776767	rs2271357	1	8	10721033	*PINX1*	G	A	0.382	A	TG (2,4)
rs12678919			8	19888502	*LPL*	A	G	0.095	A/G	TG/HDL (2,4,6)
rs10102164			8	55584167	*SOX17*	G	A	0.203	A/A	LDL/TC (2)
rs2293889			8	116668374	*TRPS1*	C	A	0.421	C	HDL (2,4)
rs2954029			8	126560154	*TRIB1*	A	T	0.459	A/A/A/T	TC/ HDL / LDL (2,4), TG (2,4,5-7)
rs9987289			8	165221337	*PPP1R3B*	G	A	0.086	G/G/G	TC / HDL/ LDL (2,4)
rs3780181			9	2630759	*VLDLR*	A	G	0.064	A/A	TC /LDL (2)
rs581080			9	39157752	*TTC39B*	G	C	0.196	G/G	HDL/TC (2,4)
rs1883025			9	106704122	*ABCA1*	G	A	0.252	G/G	TC/HDL (2,4,6)

GWAS SNP	Proxy	R2	CHR	Position in hg18	Gene	Major allele	Minor allele	MAF	Risk allele	Traits and publications
rs635634			9	116416342	*ABO*	G	A	0.190	A/A	TC / LDL(1)
rs1832007	rs6601924	1	10	5237302	*AKR1C4*	G	A	0.133	G	TG (2)
rs10904908			10	17300296	*VIM*	A	G	0.409	G	TC (2)
rs970548			10	43865874	*MARCH8*	A	C	0.262	C/C	HDL/TC (2)
rs10761731	rs10761741	0.97	10	64736192	*JMJD1C*	C	A	0.418	C	TG (2,4)
rs2068888			10	94829632	*CYP26A1*	G	A	0.468	G	TG (2,4)
rs2255141			10	113923876	*GPAM*	G	A	0.269	A/A	TC / LDL (2,4)
rs2923084			11	10345358	*AMPD3*	A	G	0.171	A	HDL (2,4)
rs10128711	rs10832962	1	11	18612847	*SPTY2D1*	A	G	0.264	A	TC (2,4)
rs7941030			11	23953727	*UBASH3B*	A	G	0.400	G/G	TC / HDL (2,4)
rs7120118			11	43918594	*NR1H3*	C	A	0.296	C	HDL (1)
rs3136441			11	46699823	*LRP4*	A	G	0.137	G	HDL (2,4)
rs7395662			11	47242866	*MADD*	G	A	0.370	A	HDL (1,4)
rs11246602			11	51368666	*OR4C46*	A	G	0.120	G	HDL (2)
rs174546			11	61326406	*FADS1-2-3*	G	A	0.339	A/G/G/G	HDL/TC/TG/LDL (2,4)
rs12801636			11	65147893	*PCNXL3*	G	A	0.231	A	HDL (2)
rs12272004	rs12292921	1	11	116127173	*BUD13*	A	C	0.075	C	TC/LDL/TG (1)
rs11603023			11	117991277	*PHLDB1*	G	A	0.416	A	TC (2)
rs11220462			11	125749162	*ST3GAL4*	G	A	0.141	A/A	TC / LDL (2,4)
rs499974			11	232920029	*MOGAT2*	C	A	0.173	C	HDL (2)
rs4883201			12	50276409	*PHC1*	A	G	0.103	A	TC (2)
rs11613352			12	56078847	*LRP1*	G	A	0.207	A/G	HDL/ TG (4)
rs7134375			12	108421917	*PDE3A*	C	A	0.442	A	HDL (2,4)
rs2338104	rs7298565	0.967	12	108421917	*UBE3B*	A	G	0.474	A	HDL (5)
rs11065987			12	110556807	*BRAP*	A	G	0.449	A/A	TC / LDL (2,4)
rs1169288			12	119901033	*HNF1A*	A	C	0.327	C/C	TC / LDL (2,4)
rs4759375	rs10773003	1	12	123030789	*SBNO1*	G	A	0.107	A	HDL (2,4)
rs838880			12	135452921	*SCARB1*	A	G	0.313	G	HDL (2,4)
rs4765127	rs11057408	1	12	228362314	*ZNF664*	C	A	0.352	A/A	HDL/TG (2,4)
rs4942486			13	104348254	*BRCA2*	G	A	0.499	A	LDL (2)
rs8017377			14	39233692	*NYNRIN*	G	A	0.457	A	LDL (2,4)
rs4983559			14	75132669	*ZBTB42*	A	G	0.379	G	HDL (2)
rs2412710			15	40471079	*CAPN3*	G	A	0.022	A	TG (2,4)
rs1532085			15	56470658	*LIPC*	G	A	0.372	A/A/A	TG/TC (2,4), HDL (1,2,4,5)
rs1800588	rs1077834	1	15	56510771	*LIPC*	A	G	0.221	A	HDL (5)
rs2652834			15	61183920	*LACTB*	G	A	0.183	G	HDL (2,4)
rs3198697			16	15037441	*PDXDC1*	G	A	0.406	G	TG (2)
rs1121980			16	52366748	*FTO*	G	A	0.424	G/A	HDL (2)/TG (2)
rs3764261			16	55550825	*CETP*	C	A	0.326	A/C/A/C	TC/TG/LDL (2,4), HDL (1,2,4,5,8)
rs16942887			16	66485543	*LCAT*	G	A	0.135	A	HDL (2,4)
rs2000999			16	70665594	*HPR*	G	A	0.183	A/A	TC / LDL (2,4)
rs2925979			16	80092291	*CMIP*	G	A	0.307	G	HDL (2,4)
rs314253			17	7032374	*DLG4*	A	G	0.315	A/A	TC /LDL (2)
rs11869286			17	35067382	*STARD3*	G	C	0.356	C	HDL (2,4)

GWAS SNP	Proxy	R2	CHR	Position in hg18	Gene	Major allele	Minor allele	MAF	Risk allele	Traits and publications
rs7206971	rs12452315	0.95	17	45413088	*C17orf157*	A	C	0.467	C/C	LDL/TC (2)
rs4148008			17	64386889	*ABCA8*	C	G	0.337	G	HDL (2,4)
rs4129767			17	73915579	*PGS1*	G	A	0.492	A	HDL (2,4)
rs8077889			17	123827546	*MPP3*	A	C	0.212	C	TG (2)
rs7240405			18	7175431	*LIPG*	G	A	0.164	G	HDL (2-4)
rs12967135	rs523288	1	18	55999349	*MC4R*	T	A	0.246	T	HDL (2,4)
rs7248104			19	8375738	*INSR*	G	A	0.417	G	TG (2)
rs10401969			19	19268718	*CILP2*	A	G	0.070	A/A/A	TC/TG (2,4), LDL (2,4,5-7)
rs492602			19	31851388	*FUT2*	A	G	0.490	G	TC (1)
rs2967605			19	38590905	*ANGPTL1*	G	A	0.181	G	HDL (1)
rs2075650			19	50087459	*TOMM40/APOE/APOC1*	A	G	0.145	G	TC (1,4)
rs157580			19	50106291	*TOMM40/APOE/APOC1*	A	G	0.388	A/G	HDL / LDL (4), TG (1)
rs439401			19	56510771	*APOC2*	G	A	0.357	G	TG (5)
rs17695224			19	57016028	*HAS1*	G	A	0.257	G	HDL (2)
rs386000			19	59484573	*LILRA3*	C	G	0.224	G	HDL (2,4)
rs731839			19	92766395	*PEPD*	A	G	0.351	G/A	TG /HDL (2)
rs6511720			19	121043296	*LDLR*	C	A	0.124	C/C	LDL (2,4,6,8), TC (2,4)
rs364585			20	12910718	*SPTLC3*	G	A	0.358	G	LDL (2)
rs2328223			20	17793921	*SNX5*	A	C	0.210	C	LDL (2)
rs2277862			20	33616196	*FER1LA*	G	A	0.146	G	TC (1)
rs2902940			20	38524901	*MAFB*	A	G	0.314	A/A	TC/LDL (2,4)
rs6065906			20	109620053	*PLTP*	A	G	0.182	A/G	HDL/TG (2,4)
rs6029526	rs6065311	1	20	139873450	*TOP1*	A	G	0.492	A/A	TC/LDL (2,4)
rs5763662			22	15295378	*MTMR3*	G	A	0.026	A	LDL (2)
rs181362			22	20262068	*UBE2L3*	G	A	0.190	G	HDL (2,4)
rs5756931			22	28708703	*PLA2G6*	A	G	0.402	A	TG (2,4)
rs138777			22	34041098	*TOM1*	G	A	0.343	A	TC (2)
rs4253772			22	45006267	*PPARA*	G	A	0.105	A/A	TC /LDL (2)

MAF: Minor allele frequency

1. Aulchenko et al. *Nat Genet* 2009

2. Global Lipids Genetics et al. *Nat Genet* 2013

3. Heid. et al. *Circ Cardiovasc Genet* 2008

4.Teslovich et al. *Nature* 2010

5. Kathiresan et al. *Nat Genet* 2009

6. Sabatti et al. *Nat Genet* 2009

7. Waterworth et al. *Arterioscler Thromb Vasc Biol* 2010

8. Willer et al. Nat Genet 2008

Supplementary Table 2. Genotype count. call rate and Hardy Weinberg Equilibrium (HWE) test for the selected SNP calculated in the FAMILY population

SNP	Minor allele	Major Allele	Genotypes	HWE* p-value	Call Rate
rs1077514	G	A	21/250/711	1.00×10^{1}	100.00
rs12027135	T	A	205/475/302	4.79×10^{-1}	100.00
rs12746810	A	G	6/133/843	6.45×10^{-1}	100.00
rs4660293	G	A	47/356/579	4.70×10^{-1}	100.00
rs11206510	G	A	35/274/673	2.71×10^{-1}	100.00
rs2479409	G	A	117/461/404	4.43×10^{-1}	99.93
rs2131925	C	A	124/414/444	7.47×10^{-2}	100.00
rs6603981	G	A	49/318/614	3.40×10^{-1}	99.87
rs646776	G	A	39/355/588	1.15×10^{-1}	100.00
rs267733	G	A	20/248/709	8.99×10^{-1}	99.34
rs12145743	C	A	109/448/425	6.18×10^{-1}	99.87
rs4650994	G	A	228/478/276	4.43×10^{-1}	100.00
rs1689800	G	A	126/470/386	3.73×10^{-1}	100.00
rs2807834	A	C	84/423/475	4.95×10^{-1}	100.00
rs4846914	G	A	150/487/345	3.20×10^{-1}	99.93
rs558971	A	G	216/480/286	6.08×10^{-1}	100.00
rs1042034	G	A	49/311/621	2.10×10^{-1}	99.93
rs1367117	A	G	96/416/470	7.66×10^{-1}	100.00
rs515135	A	G	43/287/652	1.20×10^{-1}	99.93
rs1260326	A	G	160/514/308	3.11×10^{-2}	99.93
rs6756629	A	G	5/116/861	5.90×10^{-1}	100.00
rs6544713	A	G	93/435/454	4.62×10^{-1}	100.00
rs2710642	G	A	99/434/449	7.15×10^{-1}	99.93
rs10490626	A	G	5/147/830	8.27×10^{-1}	99.93
rs2030746	A	G	144/479/359	4.61×10^{-1}	99.93
rs7570971	A	C	174/429/379	7.55×10^{-3}	100.00
rs10195252	G	A	156/479/347	6.91×10^{-1}	100.00
rs12328675	G	A	16/215/751	8.85×10^{-1}	100.00
rs2287623	G	A	170/456/356	2.60×10^{-1}	100.00
rs11694172	G	A	52/360/570	6.59×10^{-1}	100.00
rs7422339	A	C	95/408/479	5.47×10^{-1}	100.00
rs1250229	A	G	69/381/532	9.35×10^{-1}	100.00
rs2972146	C	A	128/442/412	5.78×10^{-1}	100.00
rs11563251	A	G	13/194/775	7.50×10^{-1}	100.00
rs2606736	G	A	140/478/364	4.20×10^{-1}	100.00
rs7640978	A	G	7/129/845	3.47×10^{-1}	99.93
rs2013208	A	G	224/495/261	7.49×10^{-1}	99.74
rs13326165	A	G	52/311/618	1.26×10^{-1}	99.93
rs6805251	A	G	137/503/342	2.78×10^{-2}	100.00
rs17404153	A	C	11/199/772	7.50×10^{-1}	100.00
rs645040	C	A	39/340/602	3.41×10^{-1}	99.93
rs6831256	G	A	178/497/307	3.64×10^{-1}	100.00
rs10019888	G	A	19/280/682	1.27×10^{-1}	99.93

SNP	Minor allele	Major Allele	Genotypes	HWE* p-value	Call Rate
rs442177	C	A	187/452/343	8.86×10^{-2}	100.00
rs3822072	A	G	220/488/274	9.49×10^{-1}	100.00
rs2602836	A	G	189/473/320	5.59×10^{-1}	99.93
rs13107325	A	G	7/124/851	3.21×10^{-1}	100.00
rs6450176	A	G	60/375/547	7.35×10^{-1}	100.00
rs9686661	A	G	44/318/620	6.97×10^{-1}	100.00
rs12916	G	A	168/444/370	8.32×10^{-2}	100.00
rs4530754	G	A	207/475/300	4.79×10^{-1}	100.00
rs6882076	A	G	124/475/383	2.43×10^{-1}	100.00
rs3757354	A	G	46/318/618	5.62×10^{-1}	100.00
rs2327951	G	A	71/393/518	8.09×10^{-1}	100.00
rs1800562	A	G	3/110/868	1.00×10^{1}	99.93
rs3177928	A	G	23/262/697	9.04×10^{-1}	100.00
rs2814944	A	G	29/257/696	4.06×10^{-1}	100.00
rs2758886	A	G	86/449/447	7.69×10^{-2}	100.00
rs998584	A	C	206/485/273	7.47×10^{-1}	98.17
rs3798236	G	A	133/433/407	2.97×10^{-1}	98.89
rs1936800	G	A	226/500/256	5.66×10^{-1}	100.00
rs9376090	G	A	79/348/555	2.41×10^{-2}	99.93
rs634869	A	G	190/456/336	1.17×10^{-1}	100.00
rs1564348	G	A	23/281/678	4.20×10^{-1}	99.93
rs1084651	A	G	32/244/700	7.01×10^{-2}	99.41
rs1997243	G	A	24/224/733	1.80×10^{-1}	99.93
rs702485	G	A	198/485/299	9.49×10^{-1}	100.00
rs4142995	A	C	148/479/355	5.48×10^{-1}	100.00
rs12670798	G	A	55/359/568	9.30×10^{-1}	100.00
rs4722551	G	A	21/270/691	4.05×10^{-1}	100.00
rs2072183	G	C	52/357/573	7.90×10^{-1}	99.87
rs4917014	C	A	102/417/463	6.05×10^{-1}	100.00
rs17145738	A	G	19/217/746	4.80×10^{-1}	100.00
rs38855	G	A	205/500/277	4.81×10^{-1}	100.00
rs4731702	A	G	214/514/254	1.42×10^{-1}	100.00
rs17173637	G	A	5/153/824	6.73×10^{-1}	100.00
rs9987289	A	G	10/149/822	3.06×10^{-1}	99.93
rs2271357	A	G	151/448/383	3.11×10^{-1}	100.00
rs4921914	G	A	54/325/603	2.65×10^{-1}	100.00
rs12678919	G	A	11/164/807	4.54×10^{-1}	100.00
rs10102164	A	G	46/306/630	2.77×10^{-1}	100.00
rs2293889	A	C	175/460/328	5.52×10^{-1}	97.84
rs2954029	T	A	206/490/286	8.98×10^{-1}	100.00
rs3780181	G	A	0/125/857	2.79×10^{-2}	100.00
rs581080	C	G	39/306/637	7.61×10^{-1}	100.00
rs1883025	A	G	62/371/549	1.00×10^{1}	100.00
rs635634	A	G	34/306/642	8.36×10^{-1}	100.00
rs6601924	A	G	14/234/734	4.08×10^{-1}	100.00
rs10904908	G	A	166/471/345	8.43×10^{-1}	100.00
rs970548	C	A	74/366/542	2.83×10^{-1}	100.00

SNP	Minor allele	Major Allele	Genotypes	HWE* p-value	Call Rate
rs10761741	A	C	172/475/333	8.96×10^{-1}	99.80
rs2068888	A	G	221/478/283	4.81×10^{-1}	100.00
rs2255141	A	G	78/373/531	2.92×10^{-1}	99.93
rs2923084	G	A	27/281/674	8.22×10^{-1}	100.00
rs10832962	G	A	72/375/535	5.66×10^{-1}	100.00
rs3136441	G	A	19/230/733	7.88×10^{-1}	100.00
rs7120118	G	A	87/407/488	8.78×10^{-1}	100.00
rs7395662	A	G	146/434/402	1.15×10^{-1}	100.00
rs11246602	G	A	5/222/742	5.43×10^{-3}	99.08
rs174546	A	G	108/450/424	5.22×10^{-1}	100.00
rs12801636	A	G	54/346/582	7.88×10^{-1}	100.00
rs499974	A	C	22/296/664	1.18×10^{-1}	100.00
rs12292921	C	A	10/127/845	5.89×10^{-2}	100.00
rs11603023	A	G	170/476/336	9.48×10^{-1}	100.00
rs7941030	G	A	155/476/351	7.91×10^{-1}	100.00
rs11220462	A	G	23/230/728	3.54×10^{-1}	99.80
rs4883201	G	A	11/180/791	8.62×10^{-1}	99.93
rs7134375	A	C	189/490/303	7.47×10^{-1}	100.00
rs11613352	A	G	48/310/624	2.43×10^{-1}	100.00
rs7298565	G	A	231/468/283	1.79×10^{-1}	100.00
rs11065987	G	A	209/463/310	1.39×10^{-1}	100.00
rs1169288	C	A	112/417/452	3.09×10^{-1}	99.87
rs10773003	A	G	11/189/782	1.00×10^{1}	99.93
rs11057408	A	C	114/464/404	2.94×10^{-1}	100.00
rs838880	G	A	93/428/461	7.11×10^{-1}	100.00
rs4942486	G	A	230/520/232	7.38×10^{-2}	100.00
rs8017377	A	G	196/505/281	2.75×10^{-1}	100.00
rs4983559	G	A	142/461/379	9.46×10^{-1}	100.00
rs2412710	A	G	1/41/940	3.75×10^{-1}	100.00
rs1532085	A	G	153/425/404	2.04×10^{-2}	100.00
rs1077834	G	A	56/321/605	1.37×10^{-1}	100.00
rs2652834	A	G	31/297/654	7.50×10^{-1}	100.00
rs3198697	A	G	169/460/353	3.55×10^{-1}	99.93
rs1121980	A	G	183/467/332	4.33×10^{-1}	100.00
rs3764261	A	C	113/415/454	2.18×10^{-1}	100.00
rs16942887	A	G	14/238/730	3.40×10^{-1}	100.00
rs2000999	A	G	20/312/629	9.28×10^{-3}	98.36
rs2925979	A	G	89/424/469	6.53×10^{-1}	100.00
rs314253	G	A	109/400/473	8.86×10^{-2}	100.00
rs11869286	C	G	133/434/415	2.66×10^{-1}	99.93
rs8077889	C	A	37/342/602	2.14×10^{-1}	99.93
rs12452315	C	A	218/482/282	6.54×10^{-1}	100.00
rs4148008	G	C	125/411/445	5.36×10^{-2}	99.80
rs4129767	A	G	242/483/257	6.10×10^{-1}	100.00
rs7240405	A	G	35/252/695	4.78×10^{-2}	100.00
rs523288	A	T	62/360/560	6.68×10^{-1}	100.00
rs7248104	A	G	164/491/327	3.94×10^{-1}	100.00

SNP	Minor allele	Major Allele	Genotypes	HWE* p-value	Call Rate
rs2967605	A	G	34/288/660	6.69×10^{-1}	100.00
rs6511720	A	C	14/215/753	8.83×10^{-1}	100.00
rs10401969	G	A	6/126/850	6.21×10^{-1}	99.87
rs731839	G	A	133/424/425	1.07×10^{-1}	100.00
rs157580	G	A	147/464/367	1.00×10^{1}	99.74
rs2075650	G	A	21/242/719	8.97×10^{-1}	100.00
rs439401	A	G	129/442/410	5.78×10^{-1}	99.93
rs492602	G	A	243/477/262	3.72×10^{-1}	100.00
rs17695224	A	G	56/393/532	1.55×10^{-1}	99.87
rs386000	G	C	45/344/579	5.79×10^{-1}	98.82
rs364585	A	G	129/445/408	6.77×10^{-1}	100.00
rs2328223	C	A	34/344/604	8.33×10^{-2}	100.00
rs2277862	A	G	23/240/719	6.07×10^{-1}	100.00
rs2902940	G	A	100/417/465	6.57×10^{-1}	100.00
rs6065311	G	A	236/495/251	8.48×10^{-1}	100.00
rs6065906	G	A	27/303/652	2.83×10^{-1}	99.93
rs181362	A	G	38/298/646	6.05×10^{-1}	100.00
rs5763662	A	G	4/43/935	3.12×10^{-3}	100.00
rs138777	A	G	107/460/415	2.30×10^{-1}	99.87
rs5756931	G	A	157/472/349	9.47×10^{-1}	99.54
rs4253772	A	G	12/182/788	6.13×10^{-1}	100.00

*HWE: Hardy Weinberg Equilibrium

Supplementary Table 3. Results of the child SNPs/GS on the level of LDL-cholesterol (LDL-C)

		Birth (N=456)			3y (N=421)			5y (N=361)			Mixed Model Age Adjusted		
Gene	SNP	Beta	SE	p-value	Beta	SE	p-value	Beta	SE	p-value	Beta	SE	p-value
ANGPTL3	rs2131925	0.020	0.025	4.30×10^{-1}	0.043	0.021	4.31×10^{-2}	0.041	0.023	8.36×10^{-2}	0.029	0.018	1.01×10^{-1}
ANXA9	rs267733	0.035	0.031	2.61×10^{-1}	-0.006	0.027	8.20×10^{-1}	0.017	0.029	5.70×10^{-1}	0.027	0.022	2.26×10^{-1}
CELSR2/PSRC1/SORT1	rs646776	0.054	0.029	6.16×10^{-2}	0.038	0.024	1.14×10^{-1}	0.045	0.026	8.60×10^{-2}	0.050	0.020	1.24×10^{-2}
IRF2BP2	rs558971	0.015	0.023	5.17×10^{-1}	0.028	0.018	1.35×10^{-1}	-0.018	0.020	3.71×10^{-1}	-0.001	0.016	9.56×10^{-1}
LDLRAP1	rs12027135	-0.017	0.022	4.45×10^{-1}	0.011	0.019	5.45×10^{-1}	0.046	0.021	2.74×10^{-2}	0.009	0.016	5.70×10^{-1}
MOSC1	rs2807834	0.065	0.027	1.59×10^{-2}	0.013	0.022	5.70×10^{-1}	0.016	0.024	5.04×10^{-1}	0.027	0.019	1.53×10^{-1}
PCSK9	rs11206510	0.023	0.033	4.95×10^{-1}	0.073	0.026	4.76×10^{-3}	0.022	0.029	4.46×10^{-1}	0.018	0.022	4.25×10^{-1}
PCSK9	rs2479409	-0.003	0.024	8.96×10^{-1}	0.031	0.020	1.31×10^{-1}	-0.019	0.022	3.85×10^{-1}	-0.003	0.017	8.37×10^{-1}
PIGV	rs12746810	-0.057	0.043	1.83×10^{-1}	0.042	0.035	2.35×10^{-1}	0.029	0.039	4.54×10^{-1}	-0.005	0.029	8.74×10^{-1}
ABCG5	rs6756629	-0.017	0.049	7.34×10^{-1}	0.102	0.041	1.32×10^{-2}	0.113	0.044	1.01×10^{-2}	0.073	0.034	2.94×10^{-2}
ABCG8	rs6544713	-0.037	0.027	1.72×10^{-1}	-0.049	0.021	2.08×10^{-2}	0.011	0.022	6.35×10^{-1}	-0.014	0.019	4.52×10^{-1}
APOB	rs1367117	0.041	0.025	1.03×10^{-1}	0.034	0.021	1.08×10^{-1}	0.034	0.023	1.36×10^{-1}	0.038	0.017	2.97×10^{-2}
APOB	rs515135	0.016	0.031	5.94×10^{-1}	-0.011	0.025	6.69×10^{-1}	0.002	0.028	9.35×10^{-1}	0.007	0.021	7.58×10^{-1}
EHBP1	rs2710642	0.005	0.025	8.50×10^{-1}	0.010	0.022	6.43×10^{-1}	-0.014	0.023	5.58×10^{-1}	-0.006	0.018	7.50×10^{-1}
FN1	rs1250229	-0.014	0.027	6.03×10^{-1}	-0.018	0.022	4.11×10^{-1}	0.017	0.023	4.72×10^{-1}	0.003	0.018	8.50×10^{-1}
INSIG2	rs10490626	-0.037	0.046	4.26×10^{-1}	0.007	0.039	8.53×10^{-1}	-0.027	0.045	5.57×10^{-1}	-0.016	0.033	6.27×10^{-1}
LOC84931	rs2030746	0.001	0.024	9.52×10^{-1}	-0.020	0.020	3.16×10^{-1}	-0.003	0.022	8.78×10^{-1}	-0.006	0.017	7.03×10^{-1}
UGT1A1	rs11563251	-0.033	0.038	3.81×10^{-1}	0.022	0.031	4.83×10^{-1}	0.023	0.032	4.71×10^{-1}	-0.006	0.025	8.00×10^{-1}
CMTM6	rs7640978	0.030	0.043	4.89×10^{-1}	0.022	0.037	5.61×10^{-1}	0.083	0.040	3.98×10^{-2}	0.050	0.030	9.72×10^{-2}
DNAJC13	rs17404153	-0.022	0.035	5.31×10^{-1}	-0.036	0.029	2.22×10^{-1}	-0.020	0.031	5.33×10^{-1}	-0.020	0.024	4.12×10^{-1}
DOK7	rs6831256	0.037	0.024	1.15×10^{-1}	0.061	0.019	1.62×10^{-3}	0.035	0.021	1.04×10^{-1}	0.030	0.016	6.96×10^{-2}
CSNK1G3	rs4530754	0.024	0.023	2.90×10^{-1}	0.024	0.019	2.04×10^{-1}	0.038	0.021	6.55×10^{-2}	0.023	0.016	1.46×10^{-1}
HMGCR	rs12916	0.022	0.024	3.59×10^{-1}	-0.045	0.019	1.80×10^{-2}	0.018	0.021	3.92×10^{-1}	0.009	0.016	5.90×10^{-1}
TIMD4	rs6882076	0.002	0.024	9.26×10^{-1}	0.049	0.020	1.22×10^{-2}	0.057	0.021	8.29×10^{-3}	0.028	0.016	8.66×10^{-2}
FRK	rs3798236	-0.040	0.025	1.14×10^{-1}	-0.065	0.020	1.30×10^{-3}	0.013	0.022	5.52×10^{-1}	-0.013	0.018	4.60×10^{-1}
HFE	rs1800562	0.051	0.043	2.36×10^{-1}	0.030	0.034	3.83×10^{-1}	0.060	0.037	1.08×10^{-1}	0.049	0.029	8.92×10^{-2}
HLA	rs3177928	0.023	0.034	4.86×10^{-1}	0.076	0.028	6.07×10^{-3}	0.021	0.030	4.94×10^{-1}	0.032	0.023	1.71×10^{-1}
IDOL	rs3757354	0.049	0.029	9.50×10^{-2}	0.042	0.025	9.46×10^{-2}	-0.016	0.027	5.44×10^{-1}	0.025	0.021	2.32×10^{-1}
LPA	rs1564348	-0.060	0.032	5.89×10^{-2}	0.040	0.026	1.36×10^{-1}	0.020	0.029	4.89×10^{-1}	-0.017	0.022	4.47×10^{-1}
MYLIP/GMPR	rs2327951	0.000	0.027	9.93×10^{-1}	0.061	0.022	6.47×10^{-3}	0.051	0.025	3.79×10^{-2}	0.026	0.019	1.66×10^{-1}
DNAH11	rs12670798	0.000	0.029	9.87×10^{-1}	0.028	0.024	2.39×10^{-1}	0.010	0.027	7.00×10^{-1}	0.012	0.020	5.45×10^{-1}
MIR148A	rs4722551	-0.051	0.032	1.18×10^{-1}	0.052	0.027	5.14×10^{-2}	0.030	0.029	3.03×10^{-1}	-0.009	0.022	6.87×10^{-1}
NPC1L1	rs2072183	-0.037	0.028	1.94×10^{-1}	0.017	0.023	4.65×10^{-1}	0.026	0.025	3.05×10^{-1}	-0.005	0.019	8.16×10^{-1}
PPP1R3B	rs9987289	-0.047	0.043	2.67×10^{-1}	0.013	0.038	7.24×10^{-1}	0.032	0.043	4.55×10^{-1}	0.002	0.031	9.45×10^{-1}

		Birth (N=456)			3y (N=421)			5y (N=361)			Mixed Model Age Adjusted		
Gene	SNP	Beta	SE	p-value	Beta	SE	p-value	Beta	SE	p-value	Beta	SE	p-value
SOX17	rs10102164	0.025	0.031	4.13×10^{-1}	-0.059	0.022	7.69×10^{-3}	-0.006	0.024	7.99×10^{-1}	0.016	0.021	4.38×10^{-1}
TRIB1	rs2954029	-0.015	0.025	5.41×10^{-1}	-0.031	0.020	1.29×10^{-1}	0.014	0.022	5.28×10^{-1}	0.003	0.017	8.62×10^{-1}
ABO	rs635634	-0.018	0.030	5.39×10^{-1}	0.053	0.025	3.52×10^{-2}	0.062	0.026	1.97×10^{-2}	0.017	0.020	4.16×10^{-1}
VLDLR	rs3780181	0.025	0.047	5.93×10^{-1}	0.020	0.037	5.91×10^{-1}	0.032	0.041	4.42×10^{-1}	0.016	0.031	6.14×10^{-1}
GPAM	rs2255141	-0.019	0.027	4.79×10^{-1}	-0.017	0.023	4.45×10^{-1}	-0.002	0.025	9.49×10^{-1}	-0.016	0.019	3.84×10^{-1}
BUD13	rs12292921	-0.048	0.051	3.44×10^{-1}	-0.095	0.043	2.72×10^{-2}	-0.077	0.048	1.09×10^{-1}	-0.078	0.035	2.75×10^{-2}
FADS1-2-3	rs174546	-0.015	0.024	5.27×10^{-1}	0.031	0.020	1.31×10^{-1}	0.012	0.023	5.82×10^{-1}	0.010	0.017	5.50×10^{-1}
ST3GAL4	rs11220462	-0.029	0.037	4.32×10^{-1}	0.028	0.031	3.53×10^{-1}	0.058	0.033	7.94×10^{-2}	0.009	0.025	7.09×10^{-1}
BRAP	rs11065987	-0.013	0.025	5.84×10^{-1}	-0.009	0.020	6.60×10^{-1}	-0.026	0.022	2.47×10^{-1}	-0.018	0.017	2.73×10^{-1}
HNF1A	rs1169288	0.011	0.026	6.74×10^{-1}	-0.041	0.020	3.88×10^{-2}	-0.008	0.021	7.12×10^{-1}	0.005	0.018	7.79×10^{-1}
BRCA2	rs4942486	0.037	0.023	1.11×10^{-1}	0.014	0.020	4.83×10^{-1}	0.033	0.021	1.30×10^{-1}	0.031	0.016	5.64×10^{-2}
NYNRIN	rs8017377	-0.029	0.023	2.12×10^{-1}	0.048	0.019	1.22×10^{-2}	0.042	0.020	3.89×10^{-2}	0.006	0.016	7.08×10^{-1}
CETP	rs3764261	0.021	0.026	4.04×10^{-1}	0.033	0.021	1.29×10^{-1}	0.028	0.024	2.43×10^{-1}	0.019	0.018	2.99×10^{-1}
HPR	rs2000999	-0.006	0.030	8.47×10^{-1}	0.025	0.025	3.21×10^{-1}	0.017	0.028	5.36×10^{-1}	0.004	0.021	8.33×10^{-1}
DLG4	rs314253	0.003	0.026	8.91×10^{-1}	0.054	0.021	1.05×10^{-2}	0.032	0.023	1.73×10^{-1}	0.030	0.018	9.21×10^{-2}
C17orf157	rs12452315	0.055	0.023	1.85×10^{-2}	0.010	0.020	6.14×10^{-1}	0.027	0.022	2.18×10^{-1}	0.034	0.016	3.59×10^{-2}
CILP2	rs10401969	0.030	0.048	5.26×10^{-1}	0.088	0.038	2.11×10^{-2}	0.045	0.045	3.18×10^{-1}	0.049	0.033	1.36×10^{-1}
LDLR	rs6511720	0.052	0.035	1.42×10^{-1}	0.044	0.029	1.31×10^{-1}	0.063	0.032	5.50×10^{-2}	0.057	0.024	1.80×10^{-2}
TOMM40/APOE/APOC1	rs157580	0.076	0.024	1.54×10^{-3}	0.031	0.020	1.31×10^{-1}	0.008	0.022	7.10×10^{-1}	0.039	0.017	1.86×10^{-2}
MAFB	rs2902940	0.023	0.025	3.76×10^{-1}	0.066	0.020	7.63×10^{-4}	0.025	0.021	2.45×10^{-1}	0.021	0.017	2.33×10^{-1}
SNX5	rs2328223	0.030	0.031	3.29×10^{-1}	0.037	0.025	1.44×10^{-1}	0.006	0.027	8.26×10^{-1}	0.025	0.021	2.39×10^{-1}
SPTLC3	rs364585	-0.001	0.025	9.73×10^{-1}	0.045	0.021	2.87×10^{-2}	0.024	0.023	2.87×10^{-1}	0.023	0.017	1.73×10^{-1}
TOP1	rs6065311	-0.005	0.024	8.44×10^{-1}	-0.008	0.020	6.78×10^{-1}	-0.017	0.022	4.36×10^{-1}	-0.007	0.017	6.67×10^{-1}
MTMR3	rs5763662	0.103	0.063	1.05×10^{-1}	0.057	0.052	2.77×10^{-1}	0.042	0.056	4.60×10^{-1}	0.069	0.043	1.10×10^{-1}
PPARA	rs4253772	-0.034	0.038	3.68×10^{-1}	0.016	0.032	6.27×10^{-1}	-0.018	0.034	5.98×10^{-1}	-0.030	0.026	2.57×10^{-1}
GS	GS	0.007	0.004	7.09×10^{-2}	0.019	0.003	2.40×10^{-9}	0.020	0.003	2.56×10^{-9}	0.013	0.003	2.82×10^{-7}

Beta and SE at birth, 3 year and 5 year are natural logarithm of the lipid level trait in mg/dl. The unit of beta and SE after linear mixed model is natural logarithm of the lipid level trait in mg/dl per year.

Supplementary Table 4. Results of the child SNPs/GS on the level of Total Cholesterol (TC)

		birth			3y			5y			Mixed Model Age Adjusted		
Gene	SNP	Beta	SE	p-value	Beta	SE	p-value	Beta	SE	p-value	Beta	SE	p-value
ANGPTL3	rs2131925	0.020	0.017	2.28×10^{-1}	0.024	0.014	7.53×10^{-2}	0.019	0.014	1.62×10^{-1}	0.018	0.011	1.06×10^{-1}
ASAP3	rs1077514	-0.011	0.021	6.08×10^{-1}	0.024	0.018	1.80×10^{-1}	0.009	0.018	6.15×10^{-1}	-0.006	0.015	6.86×10^{-1}
CELSR2/PSRC1/SORT1	rs646776	0.021	0.019	2.61×10^{-1}	0.020	0.015	1.98×10^{-1}	0.012	0.015	4.21×10^{-1}	0.022	0.013	8.92×10^{-2}
EVI5	rs6603981	-0.043	0.019	2.74×10^{-2}	0.008	0.015	6.02×10^{-1}	0.013	0.016	4.06×10^{-1}	-0.021	0.013	1.18×10^{-1}
IRF2BP2	rs558971	0.007	0.015	6.23×10^{-1}	0.027	0.012	2.21×10^{-2}	0.006	0.012	6.22×10^{-1}	0.005	0.010	6.06×10^{-1}
LDLRAP1	rs12027135	-0.021	0.014	1.44×10^{-1}	0.002	0.012	8.52×10^{-1}	0.019	0.012	1.26×10^{-1}	-0.003	0.010	7.95×10^{-1}
MOSC1	rs2807834	0.024	0.017	1.72×10^{-1}	0.005	0.014	7.19×10^{-1}	0.011	0.014	4.47×10^{-1}	0.008	0.012	5.28×10^{-1}
PCSK9	rs2479409	0.023	0.016	1.56×10^{-1}	0.027	0.013	3.55×10^{-2}	0.005	0.013	6.78×10^{-1}	0.013	0.011	2.19×10^{-1}
ABCB11	rs2287623	-0.004	0.015	8.08×10^{-1}	-0.004	0.013	7.51×10^{-1}	0.008	0.013	5.54×10^{-1}	-0.001	0.011	9.04×10^{-1}
ABCG5	rs6756629	0.010	0.032	7.41×10^{-1}	0.059	0.026	2.57×10^{-2}	0.059	0.026	2.32×10^{-2}	0.048	0.022	2.74×10^{-2}
ABCG8	rs6544713	0.011	0.018	5.31×10^{-1}	-0.034	0.014	1.15×10^{-2}	-0.001	0.013	9.49×10^{-1}	0.005	0.012	6.99×10^{-1}
APOB	rs1367117	0.019	0.016	2.56×10^{-1}	0.017	0.013	2.10×10^{-1}	0.010	0.014	4.80×10^{-1}	0.018	0.011	1.16×10^{-1}
FAM117B	rs11694172	-0.001	0.018	9.55×10^{-1}	0.007	0.015	6.45×10^{-1}	0.005	0.015	7.15×10^{-1}	0.000	0.013	9.84×10^{-1}
GCKR	rs1260326	-0.010	0.016	5.22×10^{-1}	-0.028	0.012	2.23×10^{-2}	0.005	0.013	7.13×10^{-1}	-0.001	0.011	9.55×10^{-1}
INSIG2	rs10490626	-0.034	0.030	2.64×10^{-1}	-0.008	0.025	7.39×10^{-1}	-0.018	0.027	4.93×10^{-1}	-0.019	0.021	3.76×10^{-1}
LOC84931	rs2030746	-0.005	0.016	7.56×10^{-1}	-0.017	0.013	1.82×10^{-1}	-0.002	0.013	8.54×10^{-1}	-0.008	0.011	4.46×10^{-1}
RAB3GAP1	rs7570971	0.003	0.016	8.41×10^{-1}	0.037	0.013	4.73×10^{-3}	0.023	0.013	6.64×10^{-2}	0.010	0.011	3.59×10^{-1}
UGT1A1	rs11563251	-0.010	0.024	6.70×10^{-1}	0.014	0.020	4.66×10^{-1}	0.008	0.019	6.65×10^{-1}	-0.001	0.016	9.62×10^{-1}
CMTM6	rs7640978	0.010	0.028	7.21×10^{-1}	0.013	0.024	5.74×10^{-1}	0.025	0.024	2.90×10^{-1}	0.022	0.020	2.56×10^{-1}
DOK7	rs6831256	0.000	0.015	9.82×10^{-1}	0.042	0.012	6.42×10^{-4}	0.033	0.012	8.36×10^{-3}	0.011	0.011	2.82×10^{-1}
CSNK1G3	rs4530754	0.004	0.015	7.82×10^{-1}	0.015	0.012	2.10×10^{-1}	0.023	0.012	5.66×10^{-2}	0.009	0.010	3.58×10^{-1}
HMGCR	rs12916	0.024	0.016	1.30×10^{-1}	-0.036	0.012	2.73×10^{-3}	-0.004	0.012	7.21×10^{-1}	0.004	0.011	6.81×10^{-1}
TIMD4	rs6882076	-0.012	0.015	4.53×10^{-1}	0.031	0.012	1.34×10^{-2}	0.028	0.013	2.89×10^{-2}	0.009	0.010	3.72×10^{-1}
FRK	rs3798236	-0.016	0.017	3.44×10^{-1}	-0.038	0.013	3.59×10^{-3}	0.002	0.013	8.66×10^{-1}	-0.001	0.011	9.25×10^{-1}
HBS1L	rs9376090	0.027	0.018	1.37×10^{-1}	-0.004	0.015	7.73×10^{-1}	0.000	0.015	9.82×10^{-1}	0.022	0.013	9.03×10^{-2}
HFE	rs1800562	0.041	0.028	1.47×10^{-1}	0.016	0.022	4.62×10^{-1}	0.034	0.022	1.18×10^{-1}	0.033	0.019	7.62×10^{-2}
HLA	rs3177928	0.003	0.022	8.75×10^{-1}	0.051	0.018	4.27×10^{-3}	0.006	0.018	7.51×10^{-1}	0.013	0.015	3.93×10^{-1}
IDOL	rs3757354	0.015	0.019	4.42×10^{-1}	0.018	0.016	2.78×10^{-1}	-0.006	0.016	7.22×10^{-1}	0.006	0.013	6.39×10^{-1}
KCNK17	rs2758886	0.004	0.016	8.23×10^{-1}	0.001	0.013	9.68×10^{-1}	-0.011	0.013	4.12×10^{-1}	-0.005	0.011	6.69×10^{-1}
LPA	rs1564348	-0.049	0.021	1.72×10^{-2}	0.023	0.017	1.78×10^{-1}	0.014	0.017	4.15×10^{-1}	-0.018	0.014	2.04×10^{-1}
C7orf50	rs1997243	-0.001	0.023	9.69×10^{-1}	0.016	0.019	4.15×10^{-1}	0.011	0.019	5.61×10^{-1}	0.006	0.016	7.04×10^{-1}
DNAH11	rs12670798	0.012	0.019	5.36×10^{-1}	0.020	0.015	1.92×10^{-1}	0.004	0.016	7.96×10^{-1}	0.010	0.013	4.18×10^{-1}
MIR148A	rs4722551	-0.076	0.021	2.50×10^{-4}	0.031	0.017	6.92×10^{-2}	0.024	0.017	1.72×10^{-1}	-0.024	0.014	9.35×10^{-2}
NPC1L1	rs2072183	-0.007	0.019	7.21×10^{-1}	0.019	0.015	2.08×10^{-1}	0.027	0.015	6.62×10^{-2}	0.009	0.013	4.94×10^{-1}

		birth			3y			5y			Mixed Model Age Adjusted		
Gene	SNP	Beta	SE	p-value	Beta	SE	p-value	Beta	SE	p-value	Beta	SE	p-value
NAT2	rs4921914	-0.037	0.018	4.37×10^{-2}	-0.030	0.015	3.94×10^{-2}	-0.021	0.015	1.54×10^{-1}	-0.023	0.012	6.94×10^{-2}
PPP1R3B	rs9987289	-0.027	0.028	3.25×10^{-1}	0.028	0.024	2.58×10^{-1}	0.027	0.025	2.85×10^{-1}	0.011	0.020	5.89×10^{-1}
SOX17	rs10102164	-0.006	0.020	7.64×10^{-1}	-0.039	0.014	5.72×10^{-3}	-0.024	0.014	8.42×10^{-2}	0.001	0.013	9.52×10^{-1}
TRIB1	rs2954029	-0.011	0.016	5.21×10^{-1}	-0.022	0.013	9.97×10^{-2}	-0.007	0.013	5.94×10^{-1}	0.000	0.011	9.95×10^{-1}
ABCA1	rs1883025	0.034	0.017	4.45×10^{-2}	0.016	0.014	2.31×10^{-1}	0.002	0.014	8.68×10^{-1}	0.015	0.012	2.07×10^{-1}
ABO	rs635634	-0.003	0.019	8.60×10^{-1}	0.036	0.016	2.54×10^{-2}	0.043	0.015	5.85×10^{-3}	0.014	0.013	2.98×10^{-1}
TTC39B	rs581080	-0.002	0.019	9.06×10^{-1}	0.039	0.016	1.20×10^{-2}	0.024	0.016	1.24×10^{-1}	0.007	0.013	5.75×10^{-1}
VLDLR	rs3780181	0.028	0.030	3.60×10^{-1}	0.014	0.023	5.56×10^{-1}	0.001	0.024	9.60×10^{-1}	0.010	0.020	6.11×10^{-1}
MARCH8	rs970548	-0.010	0.018	5.85×10^{-1}	-0.012	0.015	4.12×10^{-1}	-0.007	0.015	6.22×10^{-1}	-0.020	0.012	1.05×10^{-1}
GPAM	rs2255141	-0.018	0.018	3.04×10^{-1}	-0.008	0.015	5.71×10^{-1}	0.010	0.015	4.73×10^{-1}	-0.007	0.012	5.74×10^{-1}
VIM	rs10904908	-0.008	0.016	6.27×10^{-1}	-0.010	0.012	4.30×10^{-1}	-0.010	0.012	3.82×10^{-1}	0.003	0.011	7.60×10^{-1}
BUD13	rs12292921	-0.050	0.033	1.30×10^{-1}	-0.046	0.027	9.08×10^{-2}	-0.054	0.028	5.79×10^{-2}	-0.052	0.023	2.29×10^{-2}
FADS1-2-3	rs174546	-0.003	0.016	8.56×10^{-1}	0.025	0.013	5.77×10^{-2}	0.012	0.013	3.68×10^{-1}	0.012	0.011	2.84×10^{-1}
PHLDB1	rs11603023	-0.004	0.016	8.19×10^{-1}	0.000	0.013	9.99×10^{-1}	-0.006	0.012	6.42×10^{-1}	-0.004	0.011	7.09×10^{-1}
SPTY2D1	rs10832962	0.000	0.017	9.77×10^{-1}	0.010	0.014	5.02×10^{-1}	0.004	0.014	7.67×10^{-1}	0.011	0.012	3.33×10^{-1}
ST3GAL4	rs11220462	-0.016	0.024	4.99×10^{-1}	0.010	0.020	6.24×10^{-1}	0.031	0.019	1.17×10^{-1}	0.001	0.016	9.50×10^{-1}
UBASH3B	rs7941030	-0.009	0.015	5.74×10^{-1}	0.005	0.013	6.81×10^{-1}	-0.016	0.013	2.05×10^{-1}	-0.007	0.011	5.36×10^{-1}
BRAP	rs11065987	-0.004	0.016	7.99×10^{-1}	-0.001	0.013	9.20×10^{-1}	-0.009	0.013	4.89×10^{-1}	-0.007	0.011	5.09×10^{-1}
HNF1A	rs1169288	-0.015	0.017	3.84×10^{-1}	-0.026	0.013	4.36×10^{-2}	-0.017	0.013	1.79×10^{-1}	-0.005	0.011	6.70×10^{-1}
PHC1	rs4883201	-0.023	0.024	3.34×10^{-1}	-0.023	0.019	2.29×10^{-1}	0.002	0.020	9.27×10^{-1}	-0.017	0.016	3.09×10^{-1}
LIPC	rs1532085	-0.026	0.016	1.10×10^{-1}	0.006	0.014	6.48×10^{-1}	0.004	0.014	7.96×10^{-1}	-0.006	0.011	6.24×10^{-1}
CETP	rs3764261	0.003	0.017	8.49×10^{-1}	0.005	0.014	7.20×10^{-1}	0.014	0.014	3.13×10^{-1}	0.014	0.012	2.30×10^{-1}
HPR	rs2000999	-0.021	0.020	2.85×10^{-1}	0.024	0.016	1.37×10^{-1}	0.018	0.016	2.75×10^{-1}	-0.002	0.014	8.97×10^{-1}
DLG4	rs314253	-0.016	0.017	3.38×10^{-1}	0.036	0.013	8.47×10^{-3}	0.021	0.014	1.28×10^{-1}	0.012	0.011	2.73×10^{-1}
C17orf157	rs12452315	0.045	0.015	2.99×10^{-3}	0.007	0.013	6.05×10^{-1}	0.015	0.013	2.48×10^{-1}	0.026	0.010	1.41×10^{-2}
LIPG	rs7240405	0.000	0.021	9.95×10^{-1}	0.044	0.018	1.44×10^{-2}	0.020	0.018	2.60×10^{-1}	0.008	0.015	5.98×10^{-1}
CILP2	rs10401969	0.032	0.031	2.98×10^{-1}	0.047	0.024	5.37×10^{-2}	0.021	0.027	4.30×10^{-1}	0.031	0.021	1.39×10^{-1}
FUT2	rs492602	0.005	0.016	7.62×10^{-1}	0.025	0.013	5.51×10^{-2}	0.028	0.013	2.73×10^{-2}	0.014	0.011	1.82×10^{-1}
LDLR	rs6511720	0.026	0.023	2.66×10^{-1}	0.033	0.019	7.54×10^{-2}	0.028	0.019	1.40×10^{-1}	0.035	0.016	2.60×10^{-2}
TOMM40/APOE/APOC1	rs2075650	0.019	0.021	3.52×10^{-1}	0.073	0.017	2.13×10^{-5}	0.058	0.016	5.50×10^{-4}	0.042	0.014	3.29×10^{-3}
FER1LA	rs2277862	0.043	0.022	5.18×10^{-2}	0.049	0.018	5.72×10^{-3}	0.047	0.017	7.30×10^{-3}	0.031	0.015	4.28×10^{-2}
MAFB	rs2902940	0.010	0.017	5.27×10^{-1}	0.031	0.013	1.54×10^{-2}	0.017	0.013	1.75×10^{-1}	0.004	0.011	7.13×10^{-1}
TOP1	rs6065311	-0.007	0.016	6.42×10^{-1}	-0.007	0.013	5.90×10^{-1}	-0.026	0.013	4.59×10^{-2}	-0.012	0.011	2.87×10^{-1}
PPARA	rs4253772	-0.026	0.025	2.94×10^{-1}	0.012	0.021	5.74×10^{-1}	-0.021	0.020	2.98×10^{-1}	-0.025	0.017	1.48×10^{-1}
TOM1	rs138777	0.012	0.016	4.33×10^{-1}	-0.007	0.013	6.06×10^{-1}	-0.019	0.013	1.43×10^{-1}	-0.001	0.011	9.44×10^{-1}

		birth			3y			5y			Mixed Model Age Adjusted		
Gene	SNP	Beta	SE	p-value	Beta	SE	p-value	Beta	SE	p-value	Beta	SE	p-value
GS	GS	-0.001	0.002	7.31×10^{-1}	0.009	0.002	7.27×10^{-7}	0.009	0.002	4.07×10^{-6}	0.004	0.002	3.78×10^{-3}

Beta and SE at birth, 3 year and 5 year are natural logarithm of the lipid level trait in mg/dl. The unit of beta and SE after linear mixed model is natural logarithm of the lipid level trait in mg/dl per year.

Supplementary Table 5. Results of the child SNPs/GS on the level of HDL-cholesterol (HDL-C)

		birth			3y			5y			Mixed Model Age Adjusted		
Gene	SNP	Beta	SE	p-value	Beta	SE	p-value	Beta	SE	p-value	Beta	SE	p-value
C1orf220	rs4650994	0.003	0.021	8.76×10^{-1}	0.020	0.016	2.21×10^{-1}	-0.009	0.019	6.42×10^{-1}	0.006	0.013	6.74×10^{-1}
GALNT2	rs4846914	0.009	0.021	6.74×10^{-1}	-0.033	0.016	3.28×10^{-2}	-0.002	0.018	9.18×10^{-1}	0.005	0.013	6.91×10^{-1}
PABPC4	rs4660293	0.031	0.025	2.15×10^{-1}	-0.003	0.019	8.77×10^{-1}	-0.035	0.021	9.55×10^{-2}	0.019	0.016	2.30×10^{-1}
PIGV	rs12746810	-0.030	0.039	4.39×10^{-1}	-0.010	0.029	7.42×10^{-1}	-0.034	0.033	3.10×10^{-1}	-0.015	0.024	5.46×10^{-1}
RRNAD1	rs12145743	-0.026	0.023	2.75×10^{-1}	0.012	0.018	5.21×10^{-1}	0.033	0.020	9.98×10^{-2}	0.006	0.015	6.69×10^{-1}
ZNF648	rs1689800	0.013	0.022	5.63×10^{-1}	-0.045	0.016	6.27×10^{-3}	-0.001	0.019	9.70×10^{-1}	0.001	0.014	9.54×10^{-1}
APOB	rs1042034	0.028	0.026	2.85×10^{-1}	0.042	0.021	4.04×10^{-2}	0.039	0.023	9.01×10^{-2}	0.026	0.017	1.22×10^{-1}
COBLL1	rs12328675	0.070	0.032	2.79×10^{-2}	0.005	0.024	8.22×10^{-1}	0.013	0.027	6.29×10^{-1}	0.023	0.020	2.44×10^{-1}
CPS1	rs7422339	-0.029	0.023	2.04×10^{-1}	-0.006	0.018	7.35×10^{-1}	-0.021	0.020	2.94×10^{-1}	-0.025	0.014	7.55×10^{-2}
IRS1	rs2972146	-0.004	0.024	8.69×10^{-1}	-0.011	0.018	5.32×10^{-1}	-0.011	0.021	5.95×10^{-1}	0.003	0.015	8.34×10^{-1}
ATG7	rs2606736	-0.045	0.022	4.30×10^{-2}	0.000	0.017	9.98×10^{-1}	0.006	0.019	7.61×10^{-1}	-0.031	0.014	2.45×10^{-2}
DNAJC13	rs17404153	-0.043	0.032	1.78×10^{-1}	0.001	0.024	9.63×10^{-1}	0.007	0.027	8.01×10^{-1}	-0.009	0.020	6.32×10^{-1}
GSK3B	rs6805251	0.037	0.022	9.81×10^{-2}	-0.029	0.017	8.86×10^{-2}	-0.005	0.019	7.97×10^{-1}	0.007	0.014	5.96×10^{-1}
RBM5	rs2013208	0.020	0.021	3.58×10^{-1}	0.015	0.016	3.63×10^{-1}	0.021	0.018	2.49×10^{-1}	0.003	0.013	8.18×10^{-1}
STAB1	rs13326165	-0.001	0.025	9.72×10^{-1}	-0.017	0.019	3.91×10^{-1}	0.015	0.021	4.91×10^{-1}	-0.013	0.016	3.96×10^{-1}
ADH5	rs2602836	0.008	0.021	6.93×10^{-1}	-0.028	0.016	8.95×10^{-2}	0.010	0.018	6.04×10^{-1}	0.001	0.013	9.48×10^{-1}
C4orf52	rs10019888	0.040	0.028	1.61×10^{-1}	-0.003	0.021	8.74×10^{-1}	-0.033	0.024	1.60×10^{-1}	0.015	0.017	4.03×10^{-1}
FAM13A	rs3822072	-0.018	0.021	3.88×10^{-1}	0.033	0.015	2.73×10^{-2}	0.011	0.017	5.28×10^{-1}	-0.006	0.013	6.61×10^{-1}
SLC39A8	rs13107325	0.035	0.040	3.77×10^{-1}	-0.016	0.031	6.09×10^{-1}	-0.057	0.036	1.16×10^{-1}	0.006	0.025	8.18×10^{-1}
ARL15	rs6450176	0.025	0.024	2.86×10^{-1}	-0.044	0.018	1.32×10^{-2}	-0.027	0.020	1.78×10^{-1}	-0.002	0.015	8.92×10^{-1}
C6orf106	rs2814944	-0.016	0.030	5.93×10^{-1}	0.024	0.022	2.71×10^{-1}	-0.011	0.025	6.59×10^{-1}	-0.017	0.018	3.57×10^{-1}
CITED2	rs634869	0.021	0.021	3.28×10^{-1}	0.022	0.016	1.75×10^{-1}	0.020	0.017	2.47×10^{-1}	0.004	0.013	7.36×10^{-1}
LPA	rs1084651	-0.026	0.027	3.40×10^{-1}	-0.021	0.021	3.01×10^{-1}	-0.014	0.023	5.31×10^{-1}	-0.011	0.017	5.25×10^{-1}
RSPO3	rs1936800	0.032	0.022	1.43×10^{-1}	-0.033	0.015	3.25×10^{-2}	-0.017	0.017	3.20×10^{-1}	0.015	0.013	2.48×10^{-1}
VEGFA	rs998584	0.025	0.021	2.26×10^{-1}	-0.012	0.016	4.46×10^{-1}	0.025	0.018	1.70×10^{-1}	0.027	0.013	3.95×10^{-2}
ABP1	rs17173637	-0.024	0.039	5.43×10^{-1}	0.003	0.030	9.31×10^{-1}	-0.015	0.034	6.48×10^{-1}	-0.008	0.024	7.45×10^{-1}
DAGLB	rs702485	0.010	0.022	6.51×10^{-1}	0.020	0.017	2.43×10^{-1}	0.042	0.019	3.19×10^{-2}	0.025	0.014	7.54×10^{-2}
IKZF1	rs4917014	0.031	0.023	1.76×10^{-1}	-0.039	0.017	2.14×10^{-2}	-0.035	0.019	6.56×10^{-2}	0.016	0.014	2.56×10^{-1}
KLF14	rs4731702	0.009	0.021	6.62×10^{-1}	0.031	0.016	4.68×10^{-2}	0.010	0.017	5.83×10^{-1}	0.003	0.013	8.44×10^{-1}
MLXIPL	rs17145738	0.024	0.034	4.83×10^{-1}	-0.045	0.025	6.70×10^{-2}	-0.034	0.028	2.13×10^{-1}	0.007	0.021	7.46×10^{-1}
SNX13	rs4142995	0.032	0.022	1.46×10^{-1}	-0.017	0.017	3.21×10^{-1}	-0.009	0.019	6.51×10^{-1}	0.015	0.014	2.76×10^{-1}
LPL	rs12678919	-0.013	0.037	7.21×10^{-1}	0.005	0.028	8.65×10^{-1}	-0.006	0.033	8.64×10^{-1}	0.001	0.023	9.55×10^{-1}
PPP1R3B	rs9987289	0.015	0.039	7.07×10^{-1}	0.042	0.031	1.80×10^{-1}	0.015	0.037	6.83×10^{-1}	0.031	0.025	2.06×10^{-1}
TRIB1	rs2954029	0.035	0.023	1.25×10^{-1}	0.036	0.017	3.32×10^{-2}	0.022	0.019	2.45×10^{-1}	0.013	0.014	3.48×10^{-1}

		birth			3y			5y			Mixed Model Age Adjusted		
Gene	SNP	Beta	SE	p-value	Beta	SE	p-value	Beta	SE	p-value	Beta	SE	p-value
TRPS1	rs2293889	0.019	0.021	3.70×10^{-1}	0.038	0.015	1.29×10^{-2}	0.017	0.017	3.13×10^{-1}	0.009	0.013	5.01×10^{-1}
ABCA1	rs1883025	0.048	0.024	4.52×10^{-2}	0.052	0.018	3.20×10^{-3}	0.046	0.020	2.02×10^{-2}	0.038	0.015	8.27×10^{-3}
TTC39B	rs581080	-0.001	0.027	9.63×10^{-1}	0.045	0.020	2.51×10^{-2}	0.015	0.023	4.97×10^{-1}	0.008	0.017	6.18×10^{-1}
MARCH8	rs970548	-0.028	0.025	2.63×10^{-1}	0.012	0.019	5.17×10^{-1}	0.031	0.021	1.33×10^{-1}	-0.010	0.015	5.30×10^{-1}
AMPD3	rs2923084	-0.035	0.027	1.98×10^{-1}	0.043	0.020	3.78×10^{-2}	0.114	0.024	1.89×10^{-6}	0.015	0.017	3.69×10^{-1}
FADS1-2-3	rs174546	-0.012	0.022	5.99×10^{-1}	0.049	0.017	3.44×10^{-3}	0.026	0.019	1.69×10^{-1}	0.017	0.014	2.04×10^{-1}
LRP4	rs3136441	0.003	0.031	9.12×10^{-1}	-0.048	0.023	3.71×10^{-2}	-0.044	0.027	9.86×10^{-2}	-0.001	0.020	9.40×10^{-1}
MADD/FOLH1	rs7395662	0.010	0.022	6.52×10^{-1}	0.051	0.016	1.87×10^{-3}	0.041	0.019	3.01×10^{-2}	0.021	0.014	1.29×10^{-1}
MOGAT2	rs499974	-0.042	0.029	1.49×10^{-1}	-0.042	0.023	6.13×10^{-2}	-0.052	0.026	4.87×10^{-2}	-0.032	0.018	8.12×10^{-2}
NR1H3	rs7120118	0.020	0.023	3.95×10^{-1}	-0.001	0.017	9.47×10^{-1}	0.001	0.020	9.64×10^{-1}	0.011	0.014	4.43×10^{-1}
OR4C46	rs11246602	-0.005	0.032	8.69×10^{-1}	0.024	0.025	3.30×10^{-1}	0.024	0.028	3.96×10^{-1}	0.000	0.020	9.83×10^{-1}
PCNXL3	rs12801636	-0.028	0.026	2.84×10^{-1}	-0.036	0.019	6.93×10^{-2}	-0.053	0.022	1.73×10^{-2}	-0.027	0.016	1.02×10^{-1}
UBASH3B	rs7941030	-0.009	0.021	6.69×10^{-1}	0.018	0.016	2.65×10^{-1}	0.004	0.019	8.40×10^{-1}	0.005	0.013	7.05×10^{-1}
LRP1	rs11613352	-0.069	0.025	5.91×10^{-3}	0.002	0.019	9.06×10^{-1}	0.009	0.021	6.54×10^{-1}	-0.029	0.015	6.01×10^{-2}
MMAB/MVK	rs7298565	0.065	0.020	1.48×10^{-3}	-0.015	0.015	3.13×10^{-1}	-0.011	0.017	5.04×10^{-1}	0.026	0.013	4.10×10^{-2}
PDE3A	rs7134375	0.002	0.021	9.15×10^{-1}	0.013	0.017	4.31×10^{-1}	-0.005	0.019	7.84×10^{-1}	0.003	0.013	7.96×10^{-1}
SBNO1	rs10773003	-0.035	0.036	3.37×10^{-1}	0.025	0.027	3.52×10^{-1}	0.016	0.031	6.06×10^{-1}	-0.008	0.022	7.21×10^{-1}
SCARB1	rs838880	0.018	0.023	4.50×10^{-1}	0.008	0.018	6.32×10^{-1}	0.020	0.020	2.99×10^{-1}	0.024	0.014	8.93×10^{-2}
ZNF664	rs11057408	-0.010	0.023	6.72×10^{-1}	0.028	0.017	1.01×10^{-1}	0.012	0.019	5.20×10^{-1}	-0.005	0.014	7.06×10^{-1}
ZBTB42	rs4983559	0.005	0.022	8.16×10^{-1}	0.007	0.016	6.49×10^{-1}	-0.010	0.018	6.07×10^{-1}	0.007	0.014	6.19×10^{-1}
LACTB	rs2652834	0.032	0.027	2.37×10^{-1}	-0.027	0.020	1.95×10^{-1}	-0.002	0.022	9.24×10^{-1}	0.009	0.017	5.80×10^{-1}
LIPC	rs1077834	0.058	0.025	2.34×10^{-2}	0.072	0.019	2.32×10^{-4}	0.029	0.023	2.07×10^{-1}	0.045	0.016	4.99×10^{-3}
LIPC	rs1532085	-0.038	0.023	1.01×10^{-1}	0.009	0.018	6.09×10^{-1}	0.004	0.020	8.34×10^{-1}	-0.010	0.014	4.98×10^{-1}
CETP	rs3764261	0.047	0.023	4.12×10^{-2}	0.055	0.017	1.65×10^{-3}	0.068	0.020	7.62×10^{-4}	0.064	0.014	7.11×10^{-6}
CMIP	rs2925979	0.019	0.024	4.33×10^{-1}	-0.062	0.018	5.31×10^{-4}	-0.032	0.020	1.14×10^{-1}	-0.012	0.015	4.36×10^{-1}
FTO	rs1121980	0.005	0.021	8.02×10^{-1}	0.010	0.017	5.26×10^{-1}	-0.009	0.019	6.46×10^{-1}	-0.001	0.013	9.11×10^{-1}
LCAT	rs16942887	0.015	0.031	6.32×10^{-1}	0.048	0.024	4.55×10^{-2}	0.020	0.028	4.81×10^{-1}	0.017	0.020	3.84×10^{-1}
ABCA8	rs4148008	-0.035	0.022	1.12×10^{-1}	-0.047	0.017	5.73×10^{-3}	-0.035	0.019	6.83×10^{-2}	-0.039	0.014	4.64×10^{-3}
PGS1	rs4129767	0.064	0.022	3.54×10^{-3}	-0.036	0.016	2.61×10^{-2}	-0.027	0.018	1.32×10^{-1}	0.021	0.013	1.26×10^{-1}
STARD3	rs11869286	0.022	0.022	3.01×10^{-1}	0.008	0.016	6.40×10^{-1}	0.000	0.018	9.99×10^{-1}	0.002	0.013	8.63×10^{-1}
LIPG	rs7240405	-0.003	0.030	9.25×10^{-1}	0.023	0.023	3.26×10^{-1}	0.043	0.025	9.23×10^{-2}	0.003	0.019	8.80×10^{-1}
MC4R	rs523288	0.002	0.026	9.42×10^{-1}	0.006	0.020	7.62×10^{-1}	-0.006	0.021	7.95×10^{-1}	0.014	0.016	3.93×10^{-1}
ANGPTL4	rs2967605	0.016	0.027	5.49×10^{-1}	0.017	0.020	4.00×10^{-1}	0.036	0.023	1.20×10^{-1}	0.008	0.017	6.18×10^{-1}
FPR3	rs17695224	0.003	0.024	9.16×10^{-1}	-0.010	0.018	5.65×10^{-1}	-0.032	0.021	1.31×10^{-1}	-0.001	0.015	9.29×10^{-1}
LILRA3	rs386000	-0.012	0.026	6.32×10^{-1}	-0.008	0.019	6.84×10^{-1}	-0.007	0.022	7.40×10^{-1}	0.003	0.016	8.55×10^{-1}

		birth			3y			5y			Mixed Model Age Adjusted		
Gene	SNP	Beta	SE	p-value	Beta	SE	p-value	Beta	SE	p-value	Beta	SE	p-value
PEPD	rs731839	-0.010	0.023	6.53×10^{-1}	-0.019	0.017	2.68×10^{-1}	0.007	0.019	7.30×10^{-1}	-0.001	0.014	9.42×10^{-1}
TOMM40/APOE/APOC1	rs157580	0.003	0.022	8.78×10^{-1}	0.001	0.017	9.37×10^{-1}	-0.011	0.019	5.56×10^{-1}	0.001	0.014	9.33×10^{-1}
PLTP	rs6065906	0.001	0.027	9.67×10^{-1}	0.054	0.021	8.98×10^{-3}	0.053	0.023	2.39×10^{-2}	0.017	0.017	3.09×10^{-1}
UBE2L3	rs181362	-0.007	0.028	8.10×10^{-1}	-0.017	0.021	4.31×10^{-1}	0.015	0.024	5.32×10^{-1}	-0.003	0.017	8.77×10^{-1}
GS	GS	0.006	0.003	2.15×10^{-2}	0.003	0.002	1.59×10^{-1}	0.004	0.002	1.26×10^{-1}	0.005	0.002	3.27×10^{-3}

Beta and SE at birth, 3 year and 5 year are natural logarithm of the lipid level trait in mg/dl. The unit of beta and SE after linear mixed model is natural logarithm of the lipid level trait in mg/dl per year.

Supplementary Table 6. Results of the child SNPs/GS on the level of Triglycerides (TG)

		birth (N=456)			3y (N=421)			5y (N=361)			Mixed Model Age Adjusted		
Gene	SNP	Beta	SE	p-value	Beta	SE	p-value	Beta	SE	p-value	Beta	SE	p-value
ANGPTL3	rs2131925	0.009	0.038	8.06×10^{-1}	-0.004	0.028	8.84×10^{-1}	0.000	0.033	9.89×10^{-1}	0.011	0.022	6.17×10^{-1}
GALNT2	rs4846914	-0.029	0.035	4.10×10^{-1}	-0.003	0.025	9.04×10^{-1}	0.012	0.030	6.90×10^{-1}	0.005	0.020	8.17×10^{-1}
PIGV	rs12746810	-0.002	0.064	9.71×10^{-1}	0.068	0.047	1.45×10^{-1}	-0.010	0.055	8.53×10^{-1}	0.026	0.037	4.81×10^{-1}
APOB	rs1042034	-0.025	0.044	5.73×10^{-1}	0.069	0.033	3.72×10^{-2}	0.000	0.038	9.95×10^{-1}	0.011	0.026	6.79×10^{-1}
COBLL1	rs10195252	-0.027	0.036	4.61×10^{-1}	-0.010	0.027	7.06×10^{-1}	0.029	0.031	3.42×10^{-1}	0.002	0.021	9.42×10^{-1}
GCKR	rs1260326	0.056	0.036	1.28×10^{-1}	0.098	0.025	8.72×10^{-5}	0.025	0.030	3.98×10^{-1}	0.047	0.021	2.61×10^{-2}
IRS1	rs2972146	-0.086	0.039	2.92×10^{-2}	-0.010	0.029	7.41×10^{-1}	-0.020	0.034	5.56×10^{-1}	-0.037	0.023	1.10×10^{-1}
MSL2L1	rs645040	-0.003	0.041	9.48×10^{-1}	0.079	0.030	9.26×10^{-3}	-0.021	0.036	5.70×10^{-1}	0.010	0.024	6.90×10^{-1}
DOK7	rs6831256	-0.062	0.035	7.96×10^{-2}	-0.040	0.026	1.13×10^{-1}	0.026	0.030	3.93×10^{-1}	-0.019	0.021	3.68×10^{-1}
KLHL8	rs442177	0.029	0.036	4.16×10^{-1}	0.011	0.027	6.67×10^{-1}	0.023	0.031	4.58×10^{-1}	0.024	0.021	2.57×10^{-1}
MAP3K1	rs9686661	0.004	0.043	9.26×10^{-1}	0.090	0.031	3.31×10^{-3}	0.045	0.037	2.23×10^{-1}	0.029	0.025	2.40×10^{-1}
TIMD4	rs6882076	0.046	0.035	1.95×10^{-1}	0.022	0.026	3.91×10^{-1}	0.020	0.030	4.99×10^{-1}	0.037	0.020	7.02×10^{-2}
RSPO3	rs1936800	0.046	0.036	2.01×10^{-1}	-0.063	0.025	1.01×10^{-2}	0.027	0.029	3.50×10^{-1}	0.015	0.020	4.50×10^{-1}
VEGFA	rs998584	-0.030	0.035	3.87×10^{-1}	-0.048	0.025	5.78×10^{-2}	0.029	0.030	3.33×10^{-1}	-0.006	0.020	7.65×10^{-1}
MET	rs38855	0.035	0.034	3.15×10^{-1}	0.037	0.026	1.58×10^{-1}	0.018	0.031	5.65×10^{-1}	0.032	0.020	1.24×10^{-1}
MIR148A	rs4722551	0.018	0.048	7.12×10^{-1}	-0.058	0.035	1.00×10^{-1}	0.007	0.041	8.74×10^{-1}	-0.001	0.028	9.74×10^{-1}
MLXIPL	rs17145738	-0.011	0.056	8.49×10^{-1}	0.082	0.039	3.79×10^{-2}	-0.084	0.046	6.83×10^{-2}	-0.029	0.033	3.72×10^{-1}
LPL	rs12678919	0.005	0.062	9.34×10^{-1}	0.065	0.045	1.54×10^{-1}	0.078	0.055	1.58×10^{-1}	0.034	0.036	3.51×10^{-1}
NAT2	rs4921914	0.025	0.042	5.63×10^{-1}	-0.022	0.030	4.66×10^{-1}	-0.007	0.035	8.47×10^{-1}	-0.010	0.024	6.81×10^{-1}
PINX1	rs2271357	-0.015	0.035	6.81×10^{-1}	0.033	0.026	2.02×10^{-1}	0.013	0.031	6.63×10^{-1}	0.005	0.021	7.92×10^{-1}
TRIB1	rs2954029	0.037	0.038	3.30×10^{-1}	0.074	0.027	5.94×10^{-3}	-0.025	0.031	4.23×10^{-1}	0.015	0.022	4.92×10^{-1}
AKR1C4	rs6601924	0.078	0.051	1.28×10^{-1}	-0.035	0.036	3.35×10^{-1}	0.012	0.042	7.84×10^{-1}	0.044	0.030	1.37×10^{-1}
CYP26A1	rs2068888	-0.011	0.036	7.61×10^{-1}	-0.035	0.026	1.88×10^{-1}	-0.042	0.031	1.77×10^{-1}	-0.028	0.021	1.80×10^{-1}
JMJD1C	rs10761741	0.013	0.036	7.13×10^{-1}	0.008	0.026	7.45×10^{-1}	0.023	0.031	4.48×10^{-1}	0.014	0.021	5.05×10^{-1}
BUD13	rs12292921	0.129	0.076	9.07×10^{-2}	-0.057	0.056	3.17×10^{-1}	-0.145	0.067	3.16×10^{-2}	-0.019	0.045	6.80×10^{-1}
FADS1-2-3	rs174546	-0.032	0.036	3.77×10^{-1}	0.077	0.027	4.02×10^{-3}	0.054	0.032	8.98×10^{-2}	0.016	0.021	4.50×10^{-1}
LRP1	rs11613352	-0.043	0.041	2.99×10^{-1}	0.009	0.030	7.58×10^{-1}	0.079	0.035	2.39×10^{-2}	0.005	0.024	8.36×10^{-1}
ZNF664	rs11057408	-0.066	0.038	8.33×10^{-2}	-0.062	0.027	2.34×10^{-2}	-0.020	0.032	5.24×10^{-1}	-0.033	0.022	1.32×10^{-1}
CAPN3	rs2412710	0.099	0.107	3.55×10^{-1}	0.109	0.080	1.74×10^{-1}	0.053	0.092	5.67×10^{-1}	0.097	0.063	1.23×10^{-1}
LIPC	rs1532085	0.014	0.038	7.19×10^{-1}	-0.010	0.028	7.20×10^{-1}	0.016	0.033	6.27×10^{-1}	0.006	0.022	7.99×10^{-1}
CETP	rs3764261	0.002	0.039	9.51×10^{-1}	-0.008	0.028	7.69×10^{-1}	-0.032	0.034	3.43×10^{-1}	-0.007	0.023	7.72×10^{-1}
FTO	rs1121980	-0.027	0.035	4.37×10^{-1}	0.005	0.027	8.41×10^{-1}	-0.035	0.031	2.64×10^{-1}	-0.020	0.021	3.38×10^{-1}
PDXDC1	rs3198697	0.063	0.038	9.47×10^{-2}	-0.049	0.028	7.68×10^{-2}	-0.044	0.033	1.83×10^{-1}	0.002	0.022	9.14×10^{-1}
MPP3	rs8077889	0.047	0.043	2.68×10^{-1}	0.017	0.031	5.96×10^{-1}	0.050	0.037	1.78×10^{-1}	0.030	0.025	2.30×10^{-1}

		birth (N=456)			3y (N=421)			5y (N=361)			Mixed Model Age Adjusted		
Gene	SNP	Beta	SE	p-value	Beta	SE	p-value	Beta	SE	p-value	Beta	SE	p-value
APOC2/APOCL/APOE	rs439401	-0.043	0.036	2.34×10^{-1}	-0.017	0.025	4.96×10^{-1}	0.076	0.029	8.92×10^{-3}	0.016	0.021	4.37×10^{-1}
CILP2	rs10401969	-0.090	0.072	2.09×10^{-1}	0.146	0.050	3.40×10^{-3}	0.199	0.063	1.64×10^{-3}	0.047	0.041	2.52×10^{-1}
INSR	rs7248104	0.028	0.036	4.44×10^{-1}	0.017	0.025	4.98×10^{-1}	0.072	0.029	1.39×10^{-2}	0.057	0.021	6.65×10^{-3}
PEPD	rs731839	-0.033	0.037	3.73×10^{-1}	-0.034	0.028	2.22×10^{-1}	0.010	0.032	7.54×10^{-1}	-0.013	0.022	5.46×10^{-1}
PLTP	rs6065906	0.016	0.045	7.16×10^{-1}	0.069	0.033	3.91×10^{-2}	0.053	0.039	1.72×10^{-1}	0.022	0.027	4.11×10^{-1}
PLA2G6	rs5756931	-0.006	0.039	8.84×10^{-1}	0.017	0.027	5.36×10^{-1}	-0.020	0.032	5.33×10^{-1}	-0.016	0.022	4.83×10^{-1}
GS	GS	0.000	0.007	9.86×10^{-1}	0.010	0.005	4.37×10^{-2}	0.014	0.006	1.06×10^{-2}	0.008	0.004	3.08×10^{-2}

Beta and SE at birth, 3 year and 5 year are natural logarithm of the lipid level trait in mg/dl. The unit of beta and SE after linear mixed model is natural logarithm of the lipid level trait in mg/dl per year.

Supplementary Table 7. Age-dependent effect of the SNPs/GS showing significant results

			Birth			3y			5y			Birth to 3y		3y to 5y		Birth to 5y	
Trait	Gene	SNP	Beta	SE	p-value	Beta	SE	p-value	Beta	SE	p-value	Z-test	p-value	Z-test	p-value	Z-test	p-value
TC	*GS*	GS	0.000	0.002	8.44×10^{-1}	0.009	0.002	9.12×10^{-7}	0.009	0.002	7.71×10^{-6}	-3.242	5.94×10^{-4}	0.040	4.84×10^{-1}	-3.103	9.59×10^{-4}
HDL	*CETP*	rs3764261	0.050	0.023	3.33×10^{-2}	0.064	0.017	1.35×10^{-4}	0.075	0.020	1.93×10^{-4}	-0.501	3.08×10^{-1}	-0.407	3.42×10^{-1}	-0.813	2.08×10^{-1}
HDL	*AMPD3*	rs2923084	-0.035	0.027	1.98×10^{-1}	0.043	0.020	3.78×10^{-2}	0.114	0.024	1.89×10^{-6}	1.845	6.50×10^{-2}	-2.273	2.30×10^{-2}	-4.125	3.71×10^{-5}
LDL	*GS*	GS	0.007	0.004	6.77×10^{-2}	0.017	0.003	2.94×10^{-8}	0.020	0.003	5.72×10^{-9}	-2.084	1.86×10^{-2}	-0.743	2.29×10^{-1}	-2.641	4.13×10^{-3}

Beta and SE at birth, 3 year and 5 year are natural logarithm of the lipid level trait in mg/dl. The unit of beta and SE after linear mixed model is natural logarithm of the lipid level trait in mg/dl per year.

Supplementary Figure 1. Unadjusted power curve for LDL-cholesterol (LDL)

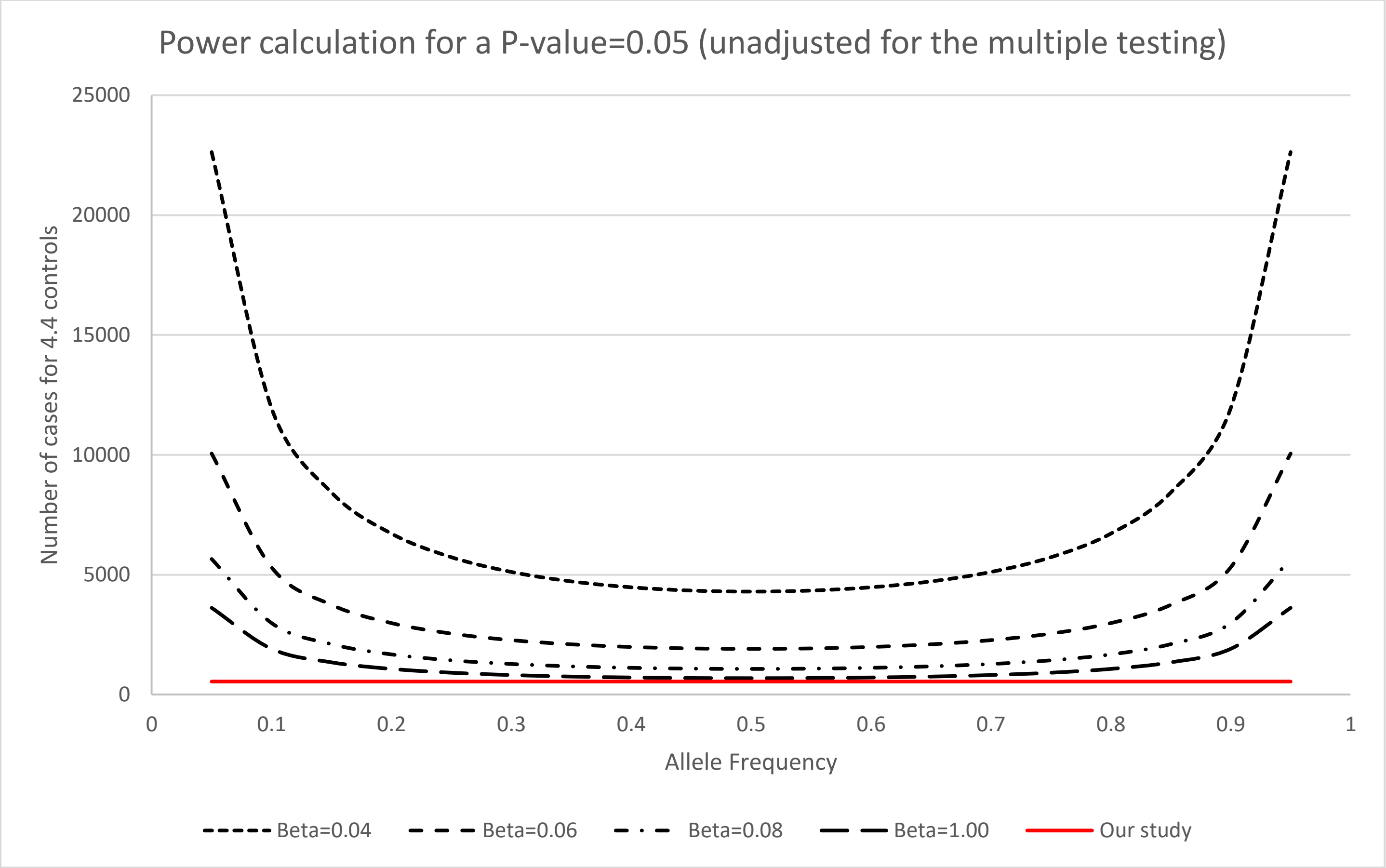

Supplementary Figure 2. Unadjusted power curve for Total Cholesterol (TC)

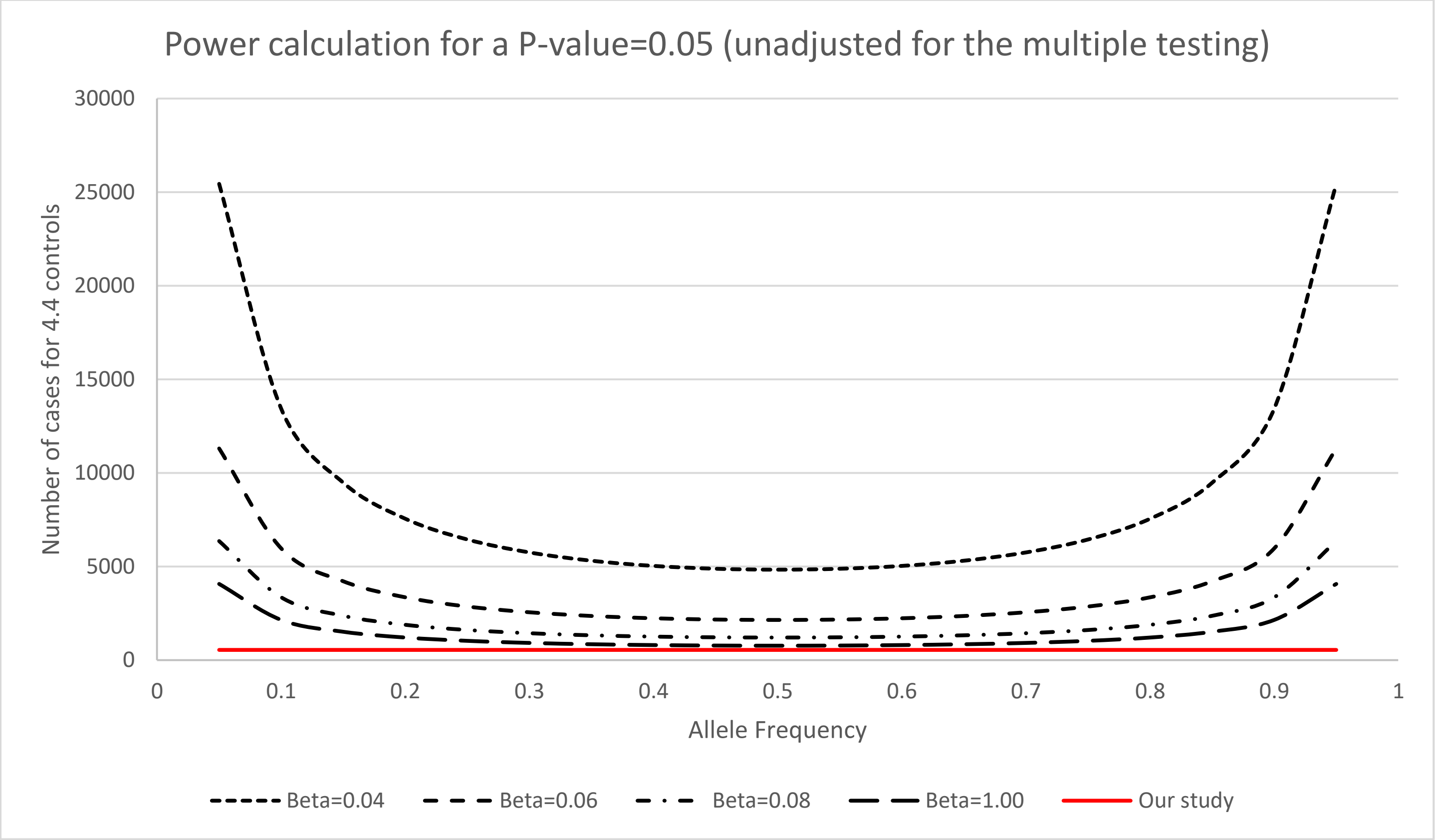

Supplementary Figure 3. Unadjusted power curve for HDL-cholesterol (HDL)

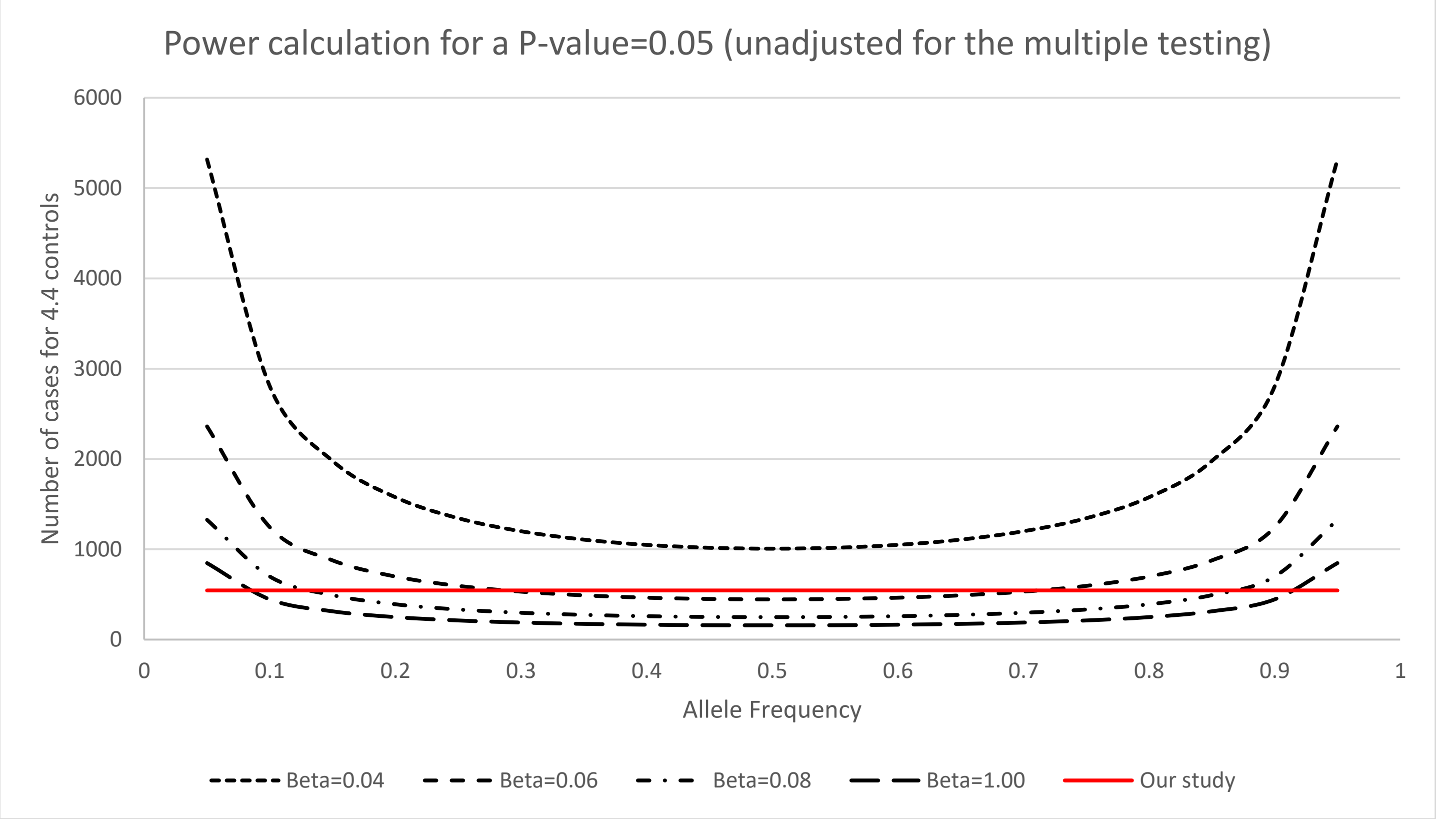

Supplementary Figure 4. Unadjusted power curve for Triglycerides (TG)

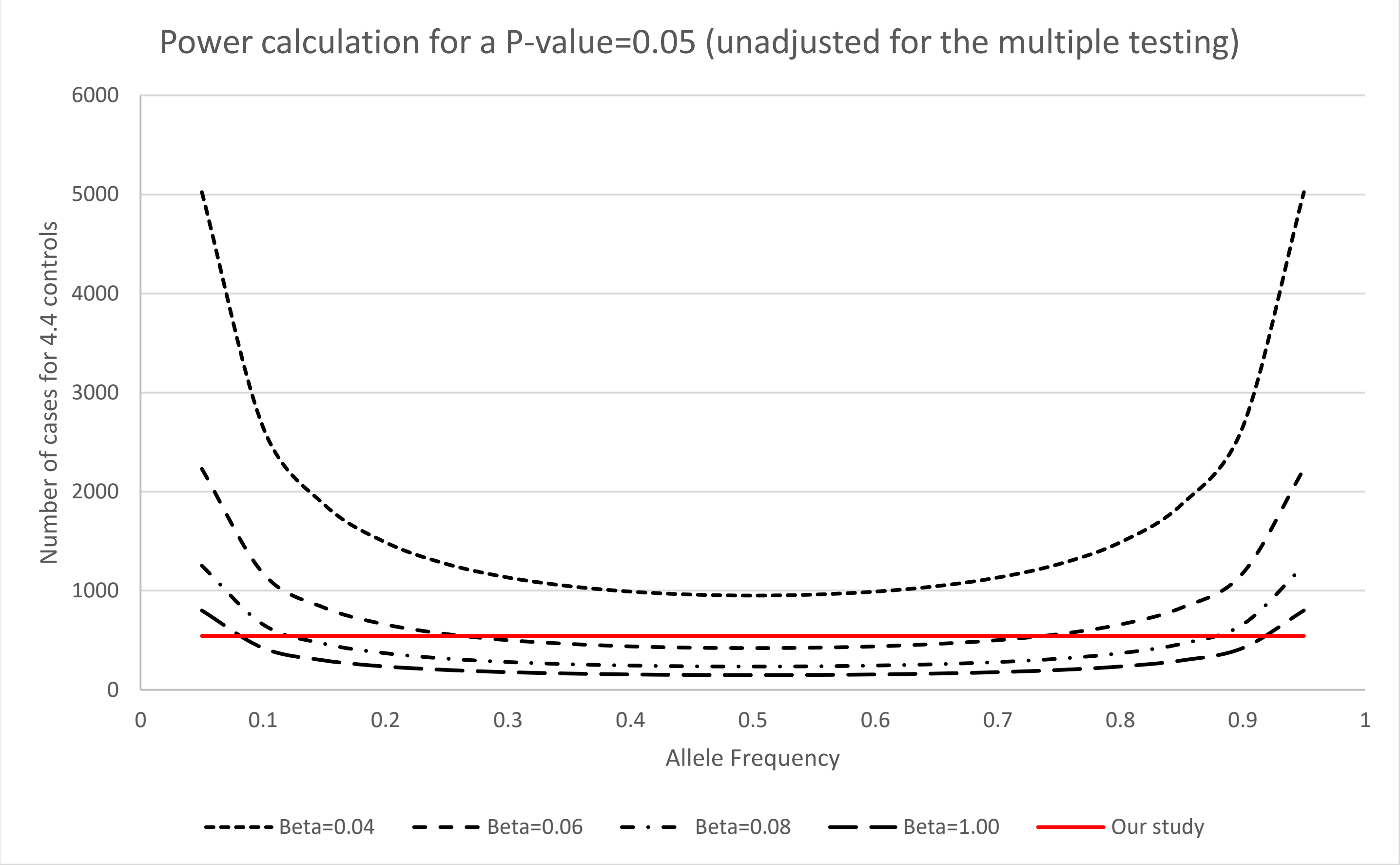

Supplementary Figure 5: Flow chart of the SNP selection

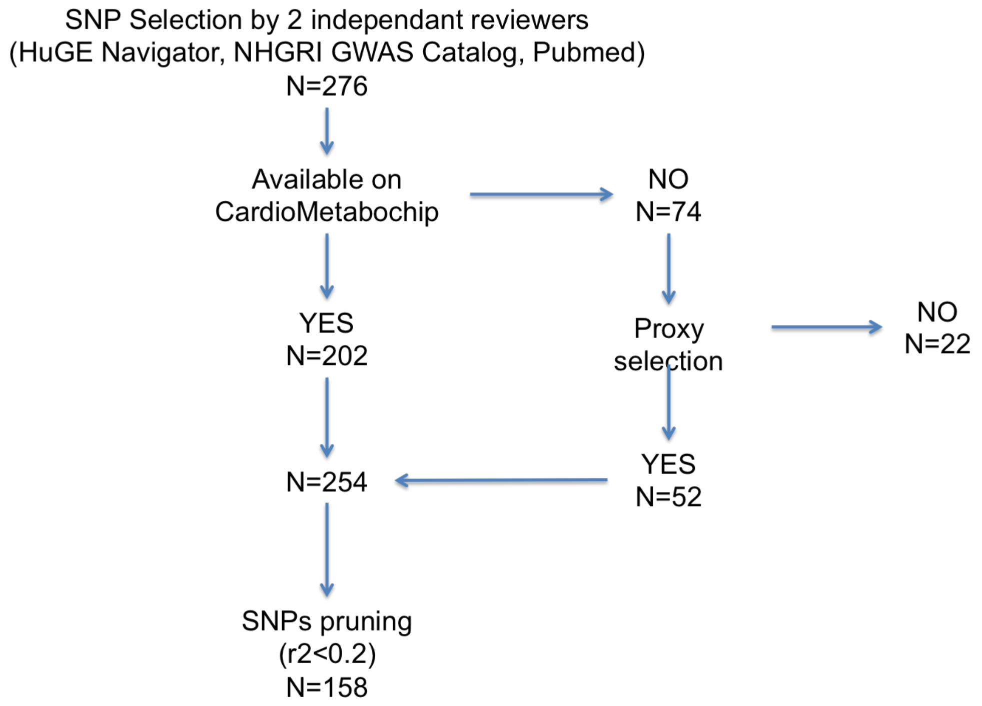

Supplementary Figure 6. Distribution of the residuals of the linear mixed effect model after transformation of the lipid level trait in mg/dL.

1) Triglyceride

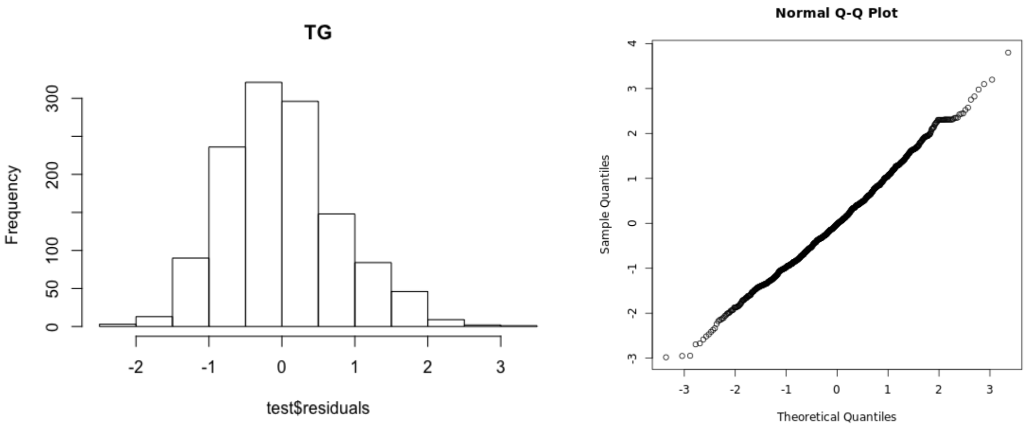

2) Total Cholesterol

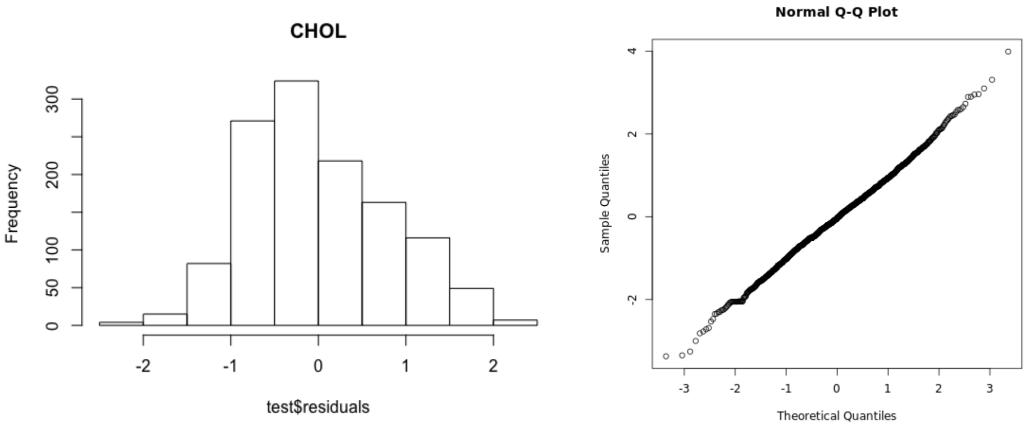

3) HDL-C

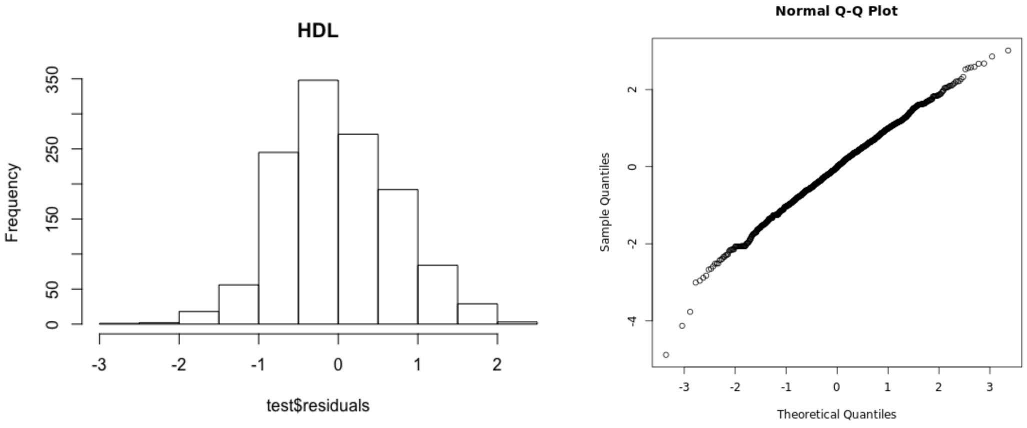

4)LDL-C

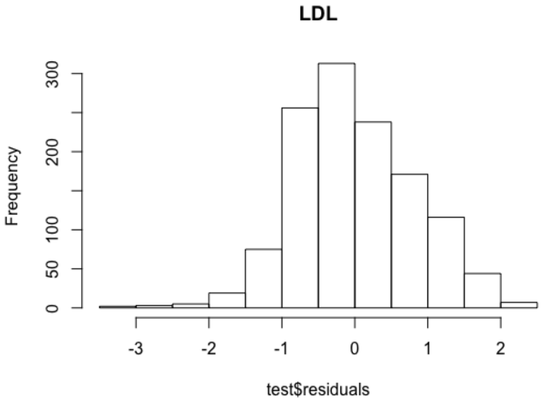

LDL
Frequency
test$residuals

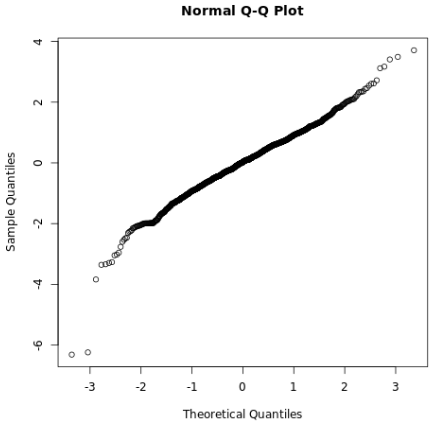

Normal Q-Q Plot
Sample Quantiles
Theoretical Quantiles
